# Relationship of Triamine-Biocide Tolerance of *Salmonella enterica* Serovar Senftenberg to Antimicrobial Susceptibility, Serum Resistance and Outer Membrane Proteins

**DOI:** 10.3390/ijms18071459

**Published:** 2017-07-11

**Authors:** Bożena Futoma-Kołoch, Bartłomiej Dudek, Katarzyna Kapczyńska, Eva Krzyżewska, Martyna Wańczyk, Kamila Korzekwa, Jacek Rybka, Elżbieta Klausa, Gabriela Bugla-Płoskońska

**Affiliations:** 1 Department of Microbiology, Institute of Genetics and Microbiology, University of Wrocław, 51-148 Wrocław, Poland; bartlomiej.dudek@uwr.edu.pl (B.D.); kamila.korzekwa@uwr.edu.pl (K.K.); wanczyk.martyna@gmail.com (W.M.); 2 Department of Immunology of Infectious Diseases, Hirszfeld Institute of Immunology and Experimental Therapy, Polish Academy of Sciences, 53-114 Wrocław, Poland; katarzyna.kapczynska@iitd.pan.wroc.pl (K.K.); eva.krzyzewska@iitd.pan.wroc.pl (E.K.); rybka@iitd.pan.wroc.pl (J.R.); 3 Regional Centre of Transfusion Medicine and Blood Bank, 50-345 Wrocław, Poland; e.klausa@rckik.wroclaw.pl

**Keywords:** *Salmonella*, biocide, serum, antimicrobial resistance, molecular biology, outer membrane protein analysis

## Abstract

A new emerging phenomenon is the association between the incorrect use of biocides in the process of disinfection in farms and the emergence of cross-resistance in *Salmonella* populations. Adaptation of the microorganisms to the sub-inhibitory concentrations of the disinfectants is not clear, but may result in an increase of sensitivity or resistance to antibiotics, depending on the biocide used and the challenged *Salmonella* serovar. Exposure of five *Salmonella enterica* subsp. *enterica* serovar Senftenberg (*S.* Senftenberg) strains to triamine-containing disinfectant did not result in variants with resistance to antibiotics, but has changed their susceptibility to normal human serum (NHS). Three biocide variants developed reduced sensitivity to NHS in comparison to the sensitive parental strains, while two isolates lost their resistance to serum. For *S.* Senftenberg, which exhibited the highest triamine tolerance (6 × MIC) and intrinsic sensitivity to 22.5% and 45% NHS, a downregulation of flagellin and enolase has been demonstrated, which might suggest a lower adhesion and virulence of the bacteria. This is the first report demonstrating the influence of biocide tolerance on NHS resistance. In conclusion, there was a potential in *S.* Senftenberg to adjust to the conditions, where the biocide containing triamine was present. However, the adaptation did not result in the increase of antibiotic resistance, but manifested in changes within outer membrane proteins’ patterns. The strategy of bacterial membrane proteins’ analysis provides an opportunity to adjust the ways of infection treatments, especially when it is connected to the life-threating bacteremia caused by *Salmonella* species.

## 1. Introduction

Cross-resistance to antibiotics of bacteria exposed to disinfectants (biocides) is an increasing problem for public health as cross-resistant phenotypes of microorganisms could potentially develop into life-threatening infections. The main reasons for increasing microbial resistance to disinfectants are mistakes during the disinfecting process itself, using chemicals that are not designed for specific microbiological pollution, inaccurate cleaning of surfaces with biocides (which causes high levels of organic matter and biofilm formation) or applying too low concentrations of biocides [1]. The implementation of hygiene supervision and standardization of the use of antibiotics and disinfectants seem to be a promising way to improve public safety [2]. There is still a lack of understanding of the mode of action of the biocides, especially when used at low or sub-inhibitory concentrations. A single exposure to some biocides has been found to be insufficient to select for multidrug-resistant (MDR) strains; however, repeated, sub-inhibitory exposure to biocides does result in the selection of MDR bacteria [3]. MDR is a major problem in the treatment of infections caused in humans by *Salmonella* isolates. It has also been noted that the drug resistance was found more frequently in the internal farm environment than in the external environment [2]. It is interesting that, although cross-resistance between biocides and antibiotics is often described for biocide-resistant mutants [2,4,5], increased susceptibility for some antimicrobials has been observed [6,7]. Moreover, resistance levels can also differ even between *Salmonella* serovars [7]. In two recent studies, we demonstrated that growth of *Salmonella enterica* subsp. *enterica* serovars Enteritidis, Typhimurium, Virchow and Zanzibar isolated from human fecal samples with sub-inhibitory concentrations of farm disinfectants containing dodecylamine (triamine) led to increased isolation of multiple antibiotic-resistant strains [8,9]. The antimicrobial efficacy of commercially-manufactured disinfectant substances (represented by quaternary ammonium salts (QAC) and QAC combined with other additives) were tested against *Salmonella* Enteritidis strains by others [7,10].

QAC and triamine-containing disinfectants are effective against many Gram-positive and Gram-negative bacteria. The antibacterial effect is caused by an increase in the permeability of the bacterial cell membrane, which leads to an osmotic imbalance and an outpouring of cytoplasm [11]. A blend containing dodecylamine-based structure was designed for the cleaning and disinfection of workplaces and devices that come into contact with food and in veterinary hygiene to disinfect animal houses (manufacturer’s data, Amity International). Exposure and further *Salmonella* adaptation to biocides may result in modification of cell envelope (an activity of efflux systems), virulence or motility [7]. It may also include various alterations of chemotaxis pathways and protein synthesis [1,12]. Several proteins have been found to be differentially expressed between biocide-tolerant variants and their parental counterparts. Recently, we have suggested that the resistance of the *S.* Typhimurium disinfectant (dodecylamine) variant to ciprofloxacin and cefotaxime was connected to the 55-kDa surface protein repression [8]. Moreover, *S.* Typhimurium and *S.* Enteritidis dodecylamine-tolerant isolates produced more surface proteins in the range of 30–40 kDa, which probably were porins OmpC (36 kDa), OmpF (35 kDa) and OmpD (34 kDa) [9]. Exposure of *Salmonella* cells to disinfectants can induce the expression of the AcrAB-TolC efflux system [13]; but after single exposure, MDR strains were not found and probably, this is not the primary mechanism of biocide tolerance generation [6]. Additionally, SugE protein is implicated in QAC resistance and is frequently found in *Salmonella* isolates of clinical and animal origin [14,15].

The majority of *Salmonella* infections result in a mild, self-limiting, gastrointestinal illness and usually do not require antimicrobial treatment. In some cases, *Salmonella* infection can develop to bacteremia in a minor subset of patients [16]. In the situation of severe enteric disease, or when *Salmonella* invades and causes bloodstream infection, therapy with antimicrobials is essential and can be life-saving. Infections with antimicrobial-resistant *Salmonella* strains resistant to first-line treatments, i.e., fluoroquinolones and cephalosporins, may cause treatment failure. There is a lack of studies regarding the susceptibility of biocide-tolerant bacteria to normal human serum (NHS), so the present work is the first study in which these aspects of bacterial virulence are discussed. Outer membrane proteins (OMPs) are described as surface virulence factors necessary for bacterial adaptation to human immune response [17]. Therefore, they have been analyzed in our research in the context of resistance to complement-mediated killing. The aim of the present study was to assess the in vitro antimicrobial effects of triamine on *S. enterica* subsp. *enterica* serovar Senftenberg sensitive to antibiotics using both MIC (minimal inhibitory concentration) and MBC (minimal bactericidal concentration) in correlation with susceptibility to NHS and OMPs patterns. Understanding the mechanisms of the individual and cross-resistance of bacteria may provide reliable clues for the design of more effective antimicrobials. 

## 2. Results

### 2.1. Salmonella enterica Tolerance to the Biocide Formulation

In this paper, the biocide formulation containing active substances triamine, ethanol, cationic surfactant and nonionic surfactant (Amity International) was used in the experiments of the generation of disinfectant-tolerant bacteria. Five *S.* Senftenberg (*Salmonella* Senftenberg) strains (131, 132, 133, 134, 135) were exposed to the disinfectant in Luria-Bertani (LB) liquid medium (Table 1). We found that the threshold for the bacterial growth was the concentration of the biocide of 8 × MIC (Minimal Inhibitory Concentration) in the LB medium, which was lethal for all tested microorganisms. The strains were grown in LB supplemented with the biocide used in the concentrations of 4 × MIC (131, 132, 134) or 6 × MIC (133, 135). After 25 days of incubation in LB containing the biocidal formulation and the following 10 days of the stability test (incubation in LB broth), the cultures were subjected to *Salmonella* spp. detection, because of the possible contamination with other microorganisms during extended passages. The isolates before and after the stability test were identified as *Salmonella* spp. on Brilliant Green agar plates as red to pink-white colonies with a red zone.

*Salmonella* variants were tested for MIC determination before and after the 10-day stability test (incubation in LB) to verify if the feature of tolerance to the biocide is stable or not. As can be seen in Table 2, MIC values were getting higher in the case of *S.* Senftenberg strains (131 ^bST^, 131 ^aST^, 132 ^bST^, 133 ^bST^, 133 ^aST^, 134 ^bST^, 134 ^aST^, 135 ^bST^) in comparison to their wild-type counterparts. Except for the *S.* Senftenberg 131 strain, in almost all tested variants cultures, MICs were decreased after the stability test, to almost the same level as it was at the beginning of the experiments. Additional MBC comparison showed that MBCs were equal to MICs for four wild tested strains: *S.* Senftenberg (131, 132, 133, 134), but not for *S.* Senftenberg 135. It was also interesting to verify if MBC levels were maintained after the test of the stability of the tolerant phenotypes. It was demonstrated that MBC did not change (strain 132 ^aST^) or was even slightly higher (131 ^aST^, 133 ^aST^, 134 ^aST^, 135 ^aST^) in comparison to the MBC value estimated for the wild-type strains. In general, both parameters MIC and MBC increased as the effect of bacterial adaptation to the increasing concentration of the biocide containing triamine.

### 2.2. Antibiotic Susceptibility Profiling

The obtained results showed that the passages of *S.* Senftenberg strains in medium containing disinfectant did not change the susceptibility pattern to antibiotics. The wild-type strains, as well as their biocide variants, were sensitive to ciprofloxacin (CIP, 5 µg), co-trimoxazole (STX, 25 µg), cefotaxime (CTX, 5 µg), amoxicillin/clavulanic acid (AMX 30; 20/10 µg) and ampicillin (AMP, 10 µg).

### 2.3. Bactericidal Activity of Human Serum against S. Senftenberg Variants Tolerant to the Disinfectant 

As C3 is a crucial protein in the activation of the serum complement cascade, standard analysis of C3 protein level in NHS used for experiments was performed. C3 concentration in NHS was 1470 mg/L, which was in the range of standard values (970–1576 mg/L for males and 1032–1495 for females). 

Bactericidal activity of diluted NHS (22.5%, 45%) was performed on *Salmonella* wild-type strains, as well as on their biocide variants. The average number of colony forming units (CFU/mL) was calculated from the colonies grown on the agar plates from the volume of 10 µL of bacterial suspensions. Between zero and 20 colonies were achieved. Two mechanisms of bacterial susceptibility to the antibacterial activity of serum were observed. Three variants of *Salmonella* strains (131 ^bST^, 131 ^aST^, 133 ^aST^, 134 ^bST^, 134 ^aST^) were found to become resistant in T_1_ or T_2_ to NHS in comparison to the sensitive parental strains, while two biocide variants (132 ^bST^, 132 ^aST^, 135 ^bST^, 135 ^aST^), lost their resistance to serum (wild-type strains were resistant) (Tables S1 and S2). In detail, the survival rate estimated for two serovars (131 and 133) increased from 10.8% (131) to 55.6% (131 ^bST^) at T_1_ in 45% NHS and to 85.3% in the case of the variant obtained after the test of stability (131 ^aST^). Survival of bacteria increased from 0.3% (parent strain 133) to 385.7% after 15 min of incubation in 45% NHS and from 15.0–103.0% at the same time in 22.5% serum. The third strain, which exhibited resistance to NHS, was 134 ^bST^, which multiplied before the test of stability in 22.5% at T_2_ (survival changed from 7.2% to 128.6%), as well as after the test of stability (76.9% survival, 134 ^aST^), at the same time, in comparison to the parent strain. In the higher concentration of serum of 45%, the same strain became resistant, as its survival raised from 5.5–70.0% at T_2_. It was interesting also to compare the feature of resistance between strains before the test of stability and after that. It was helpful to determine if the phenotype of resistance in variants was stable even if the cultures did not have any contact with the disinfectant during 10 days of incubation in LB not supplemented with the biocide. It has been observed that after the test of stability, the resistance rose (131 ^aST^, 132 ^aST^, 133 ^aST^, 134 ^aST^), or the resistance was maintained (131 ^aST^, 134 ^aST^), or vanished (132 ^aST^), depending on the time of incubation and the serum dilution. When the bactericidal activity of NHS was heat inhibited (HIS, control of experiments), bacterial cells proliferated very intensively, and all of the tested strains were resistant to 22.5% NHS (Table S1) and 45% NHS (Table S2). Regarding our results and reports of other research groups, showing that resistance to the bactericidal activity of serum is determined by OMPs [17,18,19,20], the next stage of research focused on the analysis of the protein profiles of OMPs in the context of unknown OMP-dependent tolerance to the biocide.

### 2.4. Analysis of the Two-Dimensional (2-DE) Profiles of Isolated Membrane Proteins 

**Figure 1:**
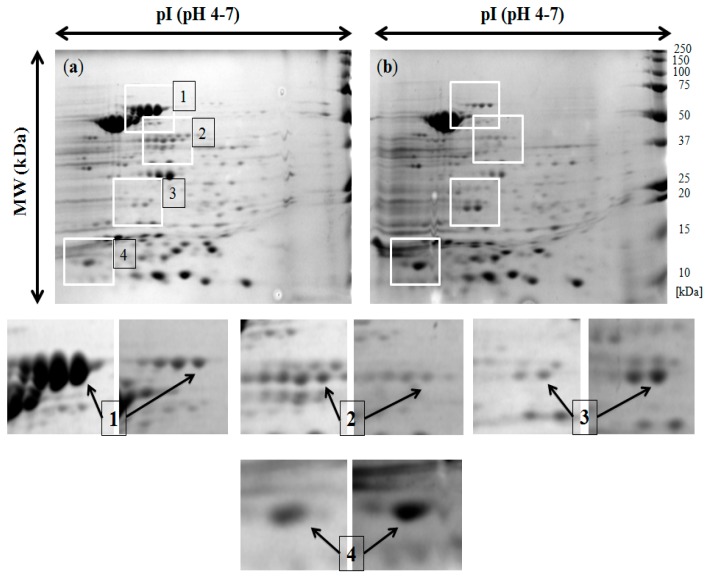
Comparative 2-D gel electrophoresis (pH 4–7) of OMPs from *Salmonella* Senftenberg 133 strain without biocide exposure (**a**) and with simultaneous resistance to triamine-containing disinfectant and NHS (**b**). Identification of flagellar protein FliC (Spot 1), enolase (Spot 2), chemotaxis response regulator protein-glutamate methylesterase (Spot 3) and outer membrane protein assembly factor (Spot 4). On the right, protein marker Precision Plus Protein™ Dual Color Standards 1610374 (Bio-Rad, Hercules, CA, USA). Left arrow refers to part (**a**), right arrow refers to part (**b**).

We applied a proteomic approach using the 2-DE and mass spectrometry analysis for the identification of specific proteins that could be involved in the phenomenon of biocide and NHS resistance of the *S.* Senftenberg 133 strain. We have chosen for this analysis *S.* Senftenberg 133 as the only strain that was primarily sensitive to NHS and belonging to the group of the highest triamine tolerance (6 × MIC). Protein spots on 2-DE were visualized within the molecular weight (MW) range of 10–250 kDa and isoelectric points (pI) of 4–7. The comparative protein pattern analysis of *S*. Senftenberg strains resistant to triamine and NHS showed differences in the presence of some proteins (Figure 1), from which four were described in detail (Table 3). MWs of identified OMPs were distributed in the range of 37.49–89.52 kDa. These research spots were distributed in the range of pI of 4.85–8.48. The detailed MASCOT search results are provided as Supporting Information. It has been noted that flagellar protein FliC (Spot 1, Figure 1), as well as enolase (Spot 2) were present in lower quantities in the biocide-tolerant variant in comparison to the wild-type parent strain. In contrast, two identified molecules, chemotaxis response regulator protein-glutamate methylesterase (Spot 3), and outer membrane protein assembly factor (Spot 4), were overproduced in the *S.* Senftenberg biocide-serum-resistant isolate, although the molecular mass of Spot 4 from 2-DE does not reflect the mass of the identified protein from the database (89.252 kDa), suggesting the degradation of the protein during the preparation process.

## 3. Discussion

*Salmonella enterica* serovars continue to be among the most important foodborne pathogens worldwide due to the significant human rates of illness reported. Public concern for the appearance of resistant zoonotic pathogens such as *Salmonella* strains to many antibiotics is challenging the poultry industry to find successful means of control [21]. The increasing use of biocides in farming, food production, hospital settings and the home is contributing to the selection of antibiotic-resistant strains [3]. Within several years, it has also been documented that biocide-resistant *Salmonella* mutants demonstrated reduced susceptibility to antibiotics or, differently, the exposure of these microorganisms to the disinfectants has not changed their sensitivity to antimicrobials. Shengzhi et al. [2] showed that 109 *Salmonella* strains were co-resistant to antibiotic and disinfectant. In inquiring research, Whitehead and co-workers [3] isolated mutants able to survive challenge with ‘‘in-use’’ concentrations of biocides after one exposure using fluorescence-activated cell sorting (FACS). These mutants were multidrug resistant and overexpressed the AcrEF efflux pump and MarA, demonstrating that biocide exposure can select for mutants with a broad, low-level antibiotic resistance. Working on *S.* Typhimurium phage type 104 (DT104) Majtánova and Majtán indicated that isolate 5551/99 represented the multiresistant phenotype, resistant to ampicillin, chloramphenicol, streptomycin and tetracycline, but the second isolate 577/99 was sensitive to all antibiotics tested [7]. Others also observed increased susceptibility of *Salmonella* for some drugs. In vitro exposure to a quaternary ammonium disinfectant containing formaldehyde and glutaraldehyde (QACFG) and triclosan led to the selection of *S.* Typhimurium cells with reduced susceptibility to several antibiotics. This was associated with overexpression of the AcrAB efflux pump and accompanied with reduced invasiveness [22]. Strains used in our study, despite the tolerance to biocide, were sensitive to antibiotics, such as ciprofloxacin, co-trimoxazole, cefotaxime, amoxicillin/clavulanic acid and ampicillin. Overall, the issue of bacterial cross-resistance needs to be clarified, but in this paper, the main characteristic that was chosen for testing was *Salmonella* sensitivity to serum.

It has been suggested that the involvement of common general responses in biocide-tolerant mutants includes several alterations in metabolic and chemotaxis pathways, protein synthesis, cell envelope or regulation of pathogenicity islands. Unlike what has been commonly reported, overexpression of AcrAB-like pumps did not seem to be the primary mechanism involved in biocide tolerance. QACs are widely used in different settings, including the food industry as a hard-surface disinfectant, antiseptic and in foaming hand sanitizers [6]. It has been known that QACs are the membrane-active agents with the target site predominantly at the cytoplasmic membrane of bacteria. Although it was found that the antibacterial efficacy of substances containing QACs with other additives was high against *S.* Enteritidis strain 85/01, it was possible to select isolates resistant to these compounds [10]. Repeated passages of *Arcobacter* spp. in a medium with a low concentration of the disinfectant Incidur, containing cationic surfactant benzalkonium chloride, increased their initial resistance to 1.5–3.5×, depending on the bacterial species or origin [5]. In our study, following several rounds of in vitro variants’ selection using increasing concentrations of triamine-containing disinfectant, *S.* Senftenberg isolates developed the biocide tolerance phenotype, with a four-fold or six-fold increase in the MIC. The test of stability relied on the incubation of the variants for 10 consecutive days in fresh LB medium without the addition of the biocide. Determination of MICs helped to conclude that the exposure of the tested strains to the triamine-containing blend resulted in increased tolerance immediately after the end of the generation of mutants that was before the stability test. However, after 10 days of incubation in non-stressful conditions, the bacteria became more sensitive to the disinfectant (Table 2). This is an optimistic phenomenon considering the public health, since tolerance to triamine was not stable. The question remains which conditions may favor stable tolerance to the biocides. The possible explanation is the presence of proteins or organic materials that reduce disinfectant activity and contribute to biofilm formation [23]. Quorum sensing is known to contribute to antibiotic resistance in *Salmonella* [24], but its role in biocide tolerance is not understood. In out further investigations, the growth of the bacteria was inhibited using the concentration of 0.04% (strains 131, 134), 0.16% (strain 132), 0.06% (strain 133) and 0.12% (strain 135). It is important to note that the bacteria were able to adapt to the increasing concentrations of the biocidal formulation, as has been previously shown [8,9].

The ability of human pathogens to survive in serum is another feature worth determining. *Salmonella* infections can result in uncomplicated diarrhea in most cases, but can lead to invasive disease [25]. Unfortunately, the mechanism of bacterial survival in NHS is not entirely understood. Considering *Salmonella* spp. surface antigens’ composition, it has been shown that long-chain LPS [26], O-antigen (O-Ag) [27], Vi capsules [28], OMPs [17,29] or the presence of fimbriae on the cell surface are virulence factors necessary for bacterial adaptation to human immune response. Recent investigations by Dudek et al. [20] revealed that sensitive *S.* Enteritidis strains possessed a high level of flagellar hook-associated protein 2 (FliD). Furthermore, others showed that O-Ag capsule-deficient mutants produced exclusively phase I flagellin (FliC) [27]. In this paper, we demonstrate that the triamine tolerant mutants displayed changes in their susceptibility profile to a diluted NHS (22.5% and 45%) when compared to their isogenic, wild-type parental strains. To our knowledge, this is the first report demonstrating the influence of biocide-tolerant phenotype to NHS-resistant pattern. *Salmonella* after repeated exposure to the biocide did not become resistant to antibiotics, but have developed resistance to NHS (Table 4). Hypothesizing, if it came to systemic infection by the bacteria with a cross-resistance to antibacterials and reduced susceptibility to serum, it would have produced treatment failure, because of an inadequate dose of a drug.

After the revision of the literature information on the role of membrane proteins in biocide or antibiotics tolerance, it can be summarized that exposure to triclosan has been associated with an upregulation of AcrAB, a major efflux system [23]. Moreover, *Salmonella* can survive challenge with in-use concentrations of some biocides; this is due to de-repression of the AcrEF efflux system, and these mutants were MDR [3]. They also included SugE, classically implicated in QACs resistance and frequently found in *Salmonella* isolates of clinical and animal origin. In this study, we compared the proteomic profile of the *S.* Senftenberg 133 variant (133 ^bST^) with the reduced susceptibility to triamine and NHS with its isogenic biocide-tolerant counterpart. We have chosen for this analysis *S.* Senftenberg 133, because it was the only one primarily sensitive to HS, belonging to the group of the highest triamine tolerance (6 × MIC). Intrinsic susceptibility of the tested serovar was essential for evaluation of 2DE analysis since sensitive *Salmonella enterica* serovars were shown to possess higher levels of flagellar hook-associated protein 2 (FliD) [20]. In our analysis, even though the variant was tolerant to the disinfectant and was sensitive to antibiotics, we have observed four distinct changes in protein patterns related to flagellin (FliC), outer membrane protein assembly factor, chemotaxis response regulator protein-glutamate methylesterase and enolase. Downregulation of flagellin and enolase factor might suggest a lower pathogenicity, including adhesion and invasion of the host cells. On the other hand, over-production of chemotaxis response regulator protein and outer membrane protein assembly factor in *S.* Senftenberg 133 ^bST^ could compensate a loss of motility. It has to be stressed that enolase is described as the multifunctional bacterial protein with the unique function of the receptor to human plasminogen. The enolase/plasminogen system is one of the mechanisms facilitating the invasiveness of pathogens, and it plays also an important role in the development of tumor tissues [30]. It seems that the tested biocide might weaken the motility-dependent virulence of *S.* Senftenberg.

In summary, there is much potential in *Salmonella* spp. to adjust to hostile environments, where the biocide containing triamine is present; however, the adaptation of the bacteria to the sub-inhibitory disinfectants’ concentrations does not always result in the increase of antibiotic resistance. In cases of reduced sensitivity of bacteria to antimicrobials, a good idea would be the use of different disinfectants alternately to minimize the risk of cross-resistance and developing of MDR phenotypes. 

## 4. Materials and Methods

### 4.1. Bacterial Strains

*Salmonella enterica* subsp. *enterica* serovar Senftenberg strains were isolated from poultry food samples in the period of November–December 2014 at the LAB-VET Veterinary Diagnostic Laboratory (Tarnowo Podgórne, Poland) by the procedures approved by Polish Centre for Accreditation. Bacterial species were serotyped in the National Serotype *Salmonella* Centre (Gdańsk, Poland). Strains used in this study were as follows: *S.* Senftenberg 131; *S.* Senftenberg 132; *S.* Senftenberg 133; *S.* Senftenberg 134; *S.* Senftenberg 135. Strains originated from the collection of the Department of Microbiology at the University of Wrocław (Wrocław, Poland). Variants before the test of stability were marked as bST and after the test of stability as aST.

### 4.2. Disinfectants and Antibiotics 

Disinfectant: commercial biocide formulation contained: triamine, 2-aminoethanol, cationic surfactants, nonionic surfactants, potassium carbonate (Amity International, Barnsley, UK) (Table 5). Antibiotics: ciprofloxacin (CIP), co-trimoxazole (STX), cefotaxime (CTX), amoxicillin/clavulanic acid (AMX 30) and ampicillin (AMP) (Thermo Fisher Scientific, Waltham, MA, USA).

### 4.3. Antimicrobial Susceptibility

Parent *S.* Senftenberg strains and their variants were tested with the broth microdilution method to determine MIC and MBC of the biocides according to Andrews et al. [31] with minor modifications. In short, biocide concentrations were prepared in Mueller-Hinton broth (Merck, Kenilworth, NJ, USA) as follows: 204.8, 102.4, 51.2, 25.6, 12.8, 6.4, 3.2, 1.6, 0.8, 0.4, 0.2, 0.1, 0.05, 0.025 µL/mL in U-bottom microtitration plates (Medlab, Raszyn, Poland). The adjustment of the bacterial precultures suspension to the density of the 0.5 McFarland standard was performed. Next, the inoculum was adjusted so that 10^4^ CFU/mL per spot were applied into the wells. The plates were incubated at 37 °C, and finally, MICs were estimated as the lowest concentration of biocide at which there was no visible growth. Either MBC was calculated. MBC was the lowest concentration that demonstrated a significant reduction (such as 99.9%) in CFU/mL when compared to the MIC dilution. The testing of bacterial susceptibility to antibiotics was done using disc diffusion and the E-test method. Data interpretation was performed according to the European Committee for Antimicrobial Susceptibility Testing (EUCAST) epidemiological cut-off values and clinical breakpoints [32]. The tests were repeated three times, including appropriate controls.

### 4.4. Isolation of Biocide Tolerant Variants and the Stability of Their Phenotypes

Isolation (generation) of variants from each culture of *Salmonella* was done according to Ricci et al. [33] and Karatzas et al. [22] (Table 1). The test was performed as previously described [8,9]. One-day precultures of the wild-type strains of *Salmonella* were exposed to subinhibitory concentrations of the disinfectant (0.5 × MIC) relevant to 0.05, 0.1, 0.2 µL/mL in dependence of the strain for 7 days; gradually increasing concentrations of the same substance (4 days for each concentration 0.75 × MIC, 1.0 × MIC, 1.25 × MIC, 1.5 × MIC); one-day incubation in LB broth (Merck) containing a 2-fold, 4-fold and 6-fold increase in biocides MICs; and ten days of incubation in LB broth, in the absence of the disinfectant to test the stability of the phenotypes. The tests of the stability of phenotypes were done on the cultures from the highest possible MICs. Three replicates of each concentration were used. Typical *Salmonella* colonies from the agar plates were transferred into sterile saline to set the density of 0.5 in McFarland standard (2 × 10^8^ cells). Then, an inoculum was created by suspending of 0.1 mL of the culture in 10 mL of saline. Next, 9.8 mL of LB medium, 0.1 mL of bacterial suspension and 0.1 mL of a given concentration of the biocide were pipetted into a sterile tube. The concentration of the biocide for each test depended on the value of the MIC estimated at the beginning of the experiments. The cultures were incubated at 37 °C for 24 h in a shaking water bath. The cultures of the bacteria were revitalized every day through the collecting of 0.1 mL bacterial suspension from the previous day’s incubation and transferring into fresh LB medium. The whole experiment, to obtain the variants tolerant to triamine-containing disinfectant, took 35 days. To confirm the presence of *Salmonella* spp. in the study, the cultures of the bacteria were inoculated onto Brilliant Green Lab-Agar (Biocorp, Warszawa, Poland).

### 4.5. Serum

NHS was obtained from Regional Centre of Transfusion Medicine and Blood Bank, Wrocław, Poland. This was conducted according to the principles expressed in the Law on public service of the blood of 20 May 2016 and in the Directive 2002/98/EC of the European Parliament and of the Council of 27 January 2003, establishing standards of quality and safety for the collection, testing, processing, storage and distribution of human blood and blood components. Blood samples were collected into aseptic tubes with clot activator and with gel for serum separation. The samples were then stored at room temperature (RT) for 30 min. After that time, the samples were centrifuged for 5 min at 4000× *g*. Only the serum samples without hemolysis and lipemia were used for experiments. The serum samples were collected, pooled and kept frozen (−70 °C) for a period no longer than 3 months. A suitable volume of serum was thawed immediately before use. Each portion was used only once. 

### 4.6. Serum C3 Concentration

The C3 concentration in the pool of NHS was quantified by a radial immunodiffusion test Human Complement C3&C4 “Nl” Bindarid^TM^ Radial Immunodiffusion Kit (The Binding Site, Birmingham, UK). C3 protein is thought to be the most important component of the C system [34]. NHS with the proper concentration of C3 glycoprotein (between 970 and 1576 mg/L) was used for these studies.

### 4.7. Serum Susceptibility Assay

The bactericidal activity of normal human serum (NHS) was determined as described previously [35] with minor modifications. It was performed in sterile polystyrene U-bottom microtitration plates (Medlab, Raszyn, Polska). *S.* Senftenberg strains and their biocide variants before and after the test of stability were subjected to the challenge of 22.5% and 45% NHS. Serum decomplemented by heating at 56 °C for 30 min (heat-inactivated normal human serum (HIS)) was used as a control [36]. After overnight incubation in LB medium (Merck, Kenilworth, NJ, USA), bacteria (500 µL) in their early exponential phase were collected by centrifugation (1500× *g* for 20 min at 4 °C). The pellet was suspended in 3 mL of phosphate-buffered saline (PBS) (POCH, Gliwice, Poland) and then diluted in the same saline to produce a suspension of approximately 10^7^ cells/mL. The volume of 20 µL of bacterial suspension and 180 µL of active or inactivated serum were transferred into the wells of the plates. Each concentration of the serum was loaded in triplicate. Finally, each well contained about 2 × 10^5^ of the bacterial cells. The mixtures were incubated at 37 °C for 0, 15 and 30 min (T_0_, T_1_ and T_2_, respectively) on a laboratory shaker with rotation at 20 rpm. Appropriate dilutions in the volume of 10 µL were then spread in triplicate onto nutrient agar plates (Biocorp, Warszawa, Poland) and incubated at 37 °C for 24 h. The average number of CFU/mL was calculated from the replicate plate counts. The survival rate for T_1_ and T_2_ was calculated as a percentage of the cell count for T_0_ (set at 100%). Strains with survival rates below 50% were considered susceptible to the bactericidal action of NHS, while those with survival rates above 50% were described as resistant to NHS. Each test was performed three times. 

### 4.8. Outer Membrane Proteins Isolation and Preparation

The isolation of OMPs was performed according to Murphy and Bartos [37] with minor modifications [20,38]. Bacterial strains were cultured overnight at 37 °C in 25 mL LB medium (Merck, Kenilworth, NJ, USA). The cells from the overnight culture were harvested (1500× *g* at 4 °C for 20 min) and suspended in 1.25 mL 1 M sodium acetate (POCH, Gliwice, Polska) with 1 mM β-mercaptoethanol (Merck). Subsequently, 11.25 mL water solution containing 5% (*w*/*v*) Zwittergent Z 3–14 (Merck, Kenilworth, NJ, USA) and 0.5 M CaCl_2_ (POCH) were added. This mixture was stirred at room temperature for 1h. To precipitate nucleic acids, 3.13 mL of 96% (*v*/*v*) cold ethanol (POCH) were added very slowly. The mixture was then centrifuged at 17,000× *g* at 4 °C for 10 min. The proteins were precipitated from the supernatant by the addition of 46.75 mL of 96% (*v*/*v*) cold ethanol and centrifuged at 17,000× *g* at 4 °C for 20 min. The pellet was left to dry at RT and then suspended in 1.5 mL 50 mM Trizma-Base (Merck) buffer, pH 8.0 containing 0.05% (*w*/*v*) Zwittergent Z 3–14 and 10 mM EDTA (Merck) and stirred at room temperature for 1 h. The solution was kept at 4 °C overnight. Insoluble material was removed by centrifugation at 12,000× *g* at 4 °C for 10 min, with OMPs present in the supernatant. Total protein concentration was measured using a commercial BCA Protein Assay Kit (Thermo Fisher Scientific, Waltham, MA, USA). 

### 4.9. Two-Dimensional Gel Electrophoresis

The OMPs were separated with 4–7 pH immobilized gradient strips (IPG 7 cm) (Bio-Rad, Hercules, CA, USA). 2-DE was carried out with the Mini-PROTEAN Tetra Cell System (Bio-Rad). Isoelectric focusing (IEF) was conducted by a stepwise increase of voltage as follows: 250 V, 20 min (linear); 4000 V, 120 min (linear); and 4000 V (rapid); until the total volt-hours reached 14 kVh. IPG strips were then loaded onto the top of 1-mm slabs comprised of a 9% polyacrylamide stacking gel and 12.5% polyacrylamide separating gel, using 0.5% agarose (Bio-Rad) with bromophenol blue dye in the running buffer. Electrophoresis was performed at 4 °C with constant power (3 W) until the dye front reached the bottom [39,40,41]. The protein spots were visualized by Coomassie Brilliant Blue (Bio-Rad). Band patterns were visualized under white light and photographed using Gel Doc™ EZ System (Bio-Rad). Image spots of proteins were analyzed by PDQuest software 8.0.1 (Bio-Rad) [20].

### 4.10. In-Gel Protein Digestion and MS Protein Identification

After isolation, 2-DE separation and staining with the Coomassie Brilliant Blue method, protein spots of interest were excised from the gel and subjected to the in-gel tryptic digestion according to the method described by Shevchenko et al. [42]. Briefly, after destaining (100 mM NH_4_HCO_3_/acetonitrile, 1:1, *v*/*v*), reduction (10 mM dithiothreitol in 100 mM NH_4_HCO_3_) and alkylation (55 mM iodoacetamide in 100 mM NH_4_HCO_3_), a suitable volume of 13 ng/µL trypsin in 10 mM ammonium bicarbonate containing 10% (*v*/*v*) acetonitrile was added to the excised gel spot cut into cubes. The obtained peptides were extracted from the gel, concentrated and desalted with the Pierce C18 tip and subjected to mass spectrometry analysis using the MALDI-TOF ultrafleXtreme instrument (Bruker, Bremen, Germany). Ten milligrams per milliliter of α-cyano-4-hydroxycinnamic acid (Bruker) in acetonitrile/0.1% TFA in H_2_O (1:1, *v*/*v*) were used as the eluent of peptides from the Pierce C18 tip directly on an MALDI plate. Spectra were acquired in positive reflector mode, averaging 2000 laser shots per MALDI-TOF spectrum. OMPs identification was achieved using a bioinformatics platform (ProteinScape 3.0., Bruker) and MASCOT (Matrix Science, 2.3.02) as a search engine to search protein sequence databases (NCBI, Swiss-Prot, date of access 10/03/2017) using the peptide mass fingerprinting method. All solvents used for digestion, MS preparation and analysis were of LC-MS grade and purchased from Merck Millipore (Billerica, MA, USA). Ammonium bicarbonate eluent additive for LC-MS, dithiothreitol and iodoacetamide were from Sigma-Aldrich (Saint Louis, MO, USA). Sequencing-grade modified trypsin was obtained from Promega (Madison, WI, USA).

## Figures and Tables

**Table 1:** Generation of triamine-tolerant *Salmonella* Senftenberg (*S.* Senftenberg) variants.

Time of Incubation	Concentration of Biocide	*S.* Senftenberg Strain				
		131	132	133	134	135
1-day preculture in LB broth	none	+	+	+	+	+
7 days in Luria-Bertani (LB) broth	0.5 × MIC	+ 0.05	+ 0.2	+ 0.05	+ 0.05	+ 0.1
Gradient 4 × 4 days in LB broth	0.75 × MIC	+ 0.075	+ 0.3	+ 0.075	+ 0.075	+ 0.15
	1.0 × MIC	+ 0.1	+ 0.4	+ 0.1	+ 0.1	+ 0.2
	1.25 × MIC	+ 0.125	+ 0.5	+ 0.125	+ 0.125	+ 0.25
	1.5 × MIC	+ 0.15	+ 0.6	+ 0.15	+ 0.15	+ 0.3
1 day in LB broth	2 × MIC	+ 0.2	+ 0.8	+ 0.2	+ 0.2	+ 0.4
	4 × MIC	+ 0.4	+ 1.6	+ 0.4	+ 0.4	+ 0.8
	6 × MIC	− 0.6	− 2.4	+ 0.6	− 0.6	+ 1.2
	8 × MIC	− 0.8	− 3.2	− 0.8	− 0.8	− 1.6
Identification on Brilliant Green	from the highest MIC (where growth was observed)	+	+	+	+	+
Stability test 10 days in LB broth	none	+	+	+	+	+
Identification on Brilliant Green	none	+	+	+	+	+

Definitions of abbreviations: (+) the growth of bacteria in broth supplemented with the biocide seen as the turbidity of the tubes contents or the presence of the colonies typical for *Salmonella* bacteria on Brilliant Green Agar; (−) no growth; concentrations of the biocide (µL/mL) are also shown. MIC, minimal inhibitory concentration.

**Table 2:** MIC and MBC values of the triamine-containing disinfectant for *Salmonella* Senftenberg strains.

Test	*S.* Senftenberg Strains														
	131	131 ^bST^	131 ^aST^	132	132 ^bST^	132 ^aST^	133	133 ^bST^	133 ^aST^	134	134 ^bST^	134 ^aST^	135	135 ^bST^	135 ^aST^
MIC (µL/mL)	0.1	0.4	0.4	0.4	1.6	0.2	0.1	0.6	0.4	0.1	0.4	0.2	0.2	0.8	0.2
MBC (µL/mL)	0.1	nt	0.4	0.4	nt	0.4	0.1	nt	0.8	0.1	nt	0.2	0.4	nt	0.8

Definitions of abbreviations: MIC, minimal inhibitory concentration; MBC, minimal bactericidal concentration; nt, not tested; bST, before the test of stability; aST, after the test of stability.

**Table 3:** Identification of isolated proteins from *Salmonella* Senftenberg 133 with resistance to both triamine-containing biocide (6 × MIC) and normal human serum (NHS).

Spots	Identified Proteins	Gene Symbols	Molecular Weight (kDa)	pI	Expression
1	Flagellin (FliC)	*fliC*	52.081	4.85	downregulated
2	Enolase	*eno*	45.628	5.25	downregulated
3	Chemotaxis response regulator protein-glutamate methylesterase	*cheB*	37.498	8.48	upregulated
4	Outer membrane protein assembly factor BamA	*bamA*	89.525	4.92	upregulated

**Table 4:** Collective phenotypic characteristic of the tested *Salmonella* Senftenberg strains and their biocide-tolerant variants.

No.	Strain	Maximal Tolerance to Biocide (See Table 1)	MIC (See Table 2)	RP in 22.5% NH (See Table S1)	RP in 45% NHS (See Table S2)	Comments
131	*S.* Senftenberg		0.1	R in T_1_	S	Resistance of the variants is maintained
131^bST^		4 × MIC	higher	R in T_1_	R in T_1_	
131^aST^			higher	R in T_1_ and T_2_	R in T_1_ and T_2_	
133	*S.* Senftenberg		0.1	S	S	Resistance of the variant is maintained
133^bST^		6 × MIC	higher	S	S	
133^aST^			higher	R in T_1_	R in T_1_	
135	*S.* Senftenberg			R in T_1_ and T_2_	R in T_1_ and T_2_	Resistance of the variants to NHS is lost in both serum concentrations
135^bST^		6 × MIC	0.2	S	S	
135^aST^			the same	S	S	
132	*S.* Senftenberg		0.4	R in T_1_	R in T_1_	Resistance of the variants to NHS is lost in higher serum concentration
132^bST^		4 × MIC	higher	R in T_2_	S	
132^aST^			lower	R in T_1_	S	
134	*S.* Senftenberg		0.1	R in T_1_	R in T_1_	Resistance of the variants is maintained
134^bST^		4 × MIC	higher	R in T_1_ and T_2_	R in T_1_	
134^aST^			higher	R in T_1_ and T_2_	R in T_1_ and T_2_	

Definitions of abbreviations: MIC, minimal inhibitory concentration; NHS, normal human serum; HIS, heat-inactivated normal human serum; RP, resistance pattern; S, sensitive; R, resistant; bST, before the test of stability; aST, after the test of stability.

**Table 5:** Veterinary industry and healthcare environment biocide formulations used in this study (according to the manufacturers’ instructions).

Active Substances	Recommended Contact Time	Experimental Contact Time (See Table 1)	Recommended Working Concentration	Experimental Working Concentration	Mechanisms of Action Against Bacteria
triamine, 2-aminoethanol, cationic surfactants, nonionic surfactants	5–10 min	24 days	(2.5%) 2.5 mL/100 mL	From 5 µL/100 mL (0.005%) to 320 µL/100 mL (0.32%)	penetration of outer membrane of bacterial cell disrupting of RNA of the microorganism preventing of replication of DNA

## Supplementary Materials

Supplementary materials can be found at www.mdpi.com/1422-0067/18/7/1459/s1.

## Abbreviations

aST	After the test of stability
bST	Before the test of stability
CFU	Colony-forming units
h	Hours
HIS	Heat-inactivated normal human serum
MDR	Multidrug-resistant
MIC	Minimal inhibitory concentration
MBC	Minimal bactericidal concentration
NHS	Normal human serum
OMPs	Outer membrane proteins
RP	Resistance pattern
QAC	Quaternary ammonium salts
R	Resistant
S	Sensitive
2-DE	Two-dimensional gel electrophoresis
