# Supplementary material for: Relationship of Triamine-Biocide Tolerance of Salmonella enterica Serovar Senftenberg to Antimicrobial Susceptibility, Serum Resistance and Outer Membrane Proteins
Source: Int J Mol Sci. 2017 Jul 11;18(7):1459. doi: 10.3390/ijms18071459 (PMC5535950; doi:10.3390/ijms18071459)
Supplement: Supplementary file 1 [file ijms-18-01459-s001.zip › ijms-205418-supplementary/S3_File.pdf]

Mascot Search Results

Protein View

Match to: **FLIC_SALCH** Score: **101**
Flagellin OS=Salmonella choleraesuis (strain SC-B67) GN=fliC PE=3 SV=3
Found in search of 281474976711690.mgf

Nominal mass (M_r): **52081**; Calculated pI value: **4.85**
NCBI BLAST search of FLIC_SALCH against nr
Unformatted sequence string for pasting into other applications

Taxonomy: Salmonella enterica subsp. enterica serovar Choleraesuis str. SC-B67

Variable modifications: Carboxymethyl (C),Oxidation (M)
Cleavage by Trypsin: cuts C-term side of KR unless next residue is P
Sequence Coverage: **6%**

Matched peptides shown in **Bold Red**

1 MAQVINTNSL SLLTQNNLNK SQSALGTAIE RLSSGLRINS AK**DDAAGQAI**
51 **ANR**FTANIKG LTQASRNAND GISIAQTTEG ALNEINNNLQ RVRELAVQSA
101 NSTNSQSDLD SIQAEITQRL NEIDR**VSGQT** **QFNGVK**VLAQ DNTLTIQVGA
151 NDGETIDIDL KQINSQTLGL DTLNVQKKYD VSDTAVAASY SDSKQNIAVP
201 DKTAITAKIG AATSGGAGIK ADISFKDGKY YATVSGYDDA ADTDKNGTYE
251 VTVAADTGAV TFATTPTVVD LPTDAKAVSK VQQNDTEIAA TNAKAALKAA
301 GVADAEADTA TLVKMSYTDN NGKVIDGGFA FKTSGGYYAA SVDKSGAASL
351 KVTSYVDATT GTEKTAANKL GGADGKTEVV TIDGKTYNAS KAAGHNFKAQ
401 PELAEAAATT TENPLQKIDA ALAQVDALR**S** **DLGAVQNR**FN SAITNLGNTV
451 NNLSSARSRI EDSDYATEVS NMSRAQILQQ AGTSVLAQAN QVPQNVLSLL
501 R

Show predicted peptides also

Sort Peptides By ◉ Residue Number ○ Increasing Mass ○ Decreasing Mass

Start - End	Observed	Mr(expt)	Mr(calc)	ppm	Miss	Sequence	
43 - 53	1101.5524	1100.5451	1100.5210	22	0	K.DDAAGQAIANR.F	(Ions score 58)
126 - 136	1164.6100	1163.6027	1163.5935	8	0	R.VSGQTQFNGVK.V	(Ions score 18)
430 - 438	959.5074	958.5001	958.4832	18	0	R.SDLGAVQNR.F	(Ions score 25)

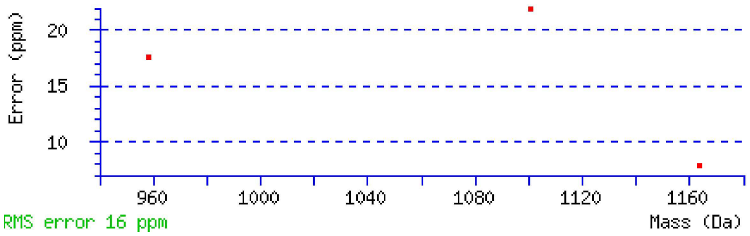

```
ID   FLIC_SALCH              Reviewed;         501 AA.
AC   P06176; Q57N43;
DT   01-JAN-1988, integrated into UniProtKB/Swiss-Prot.
DT   23-JAN-2007, sequence version 3.
DT   16-MAY-2012, entry version 86.
DE   RecName: Full=Flagellin;
DE   AltName: Full=Phase 1-C flagellin;
GN   Name=fliC; OrderedLocusNames=SCH_1962;
OS   Salmonella choleraesuis (strain SC-B67).
OC   Bacteria; Proteobacteria; Gammaproteobacteria; Enterobacteriales;
OC   Enterobacteriaceae; Salmonella;
OC   Salmonella enterica subsp. enterica serovar Choleraesuis.
OX   NCBI_TaxID=321314;
RN   [1]
RP   NUCLEOTIDE SEQUENCE [GENOMIC DNA].
RC   STRAIN=ATCC 7001;
RX   MEDLINE=86143833; PubMed=4093985; DOI=10.1016/0022-2836(85)90397-3;
```

```
RA   Wei L.-N., Joys T.M.;
RT   "Covalent structure of three phase-1 flagellar filament proteins of
RT   Salmonella.";
RL   J. Mol. Biol. 186:791-803(1985).
RN   [2]
RP   NUCLEOTIDE SEQUENCE [LARGE SCALE GENOMIC DNA].
RC   STRAIN=SC-B67;
RX   PubMed=15781495; DOI=10.1093/nar/gki297;
RA   Chiu C.-H., Tang P., Chu C., Hu S., Bao Q., Yu J., Chou Y.-Y.,
RA   Wang H.-S., Lee Y.-S.;
RT   "The genome sequence of Salmonella enterica serovar Choleraesuis, a
RT   highly invasive and resistant zoonotic pathogen.";
RL   Nucleic Acids Res. 33:1690-1698(2005).
CC   -!- FUNCTION: Flagellin is the subunit protein which polymerizes to
CC       form the filaments of bacterial flagella.
CC   -!- SUBCELLULAR LOCATION: Secreted. Bacterial flagellum.
CC   -!- MISCELLANEOUS: Individual Salmonella serotypes usually alternate
CC       between the production of 2 antigenic forms of flagella, termed
CC       phase 1 and phase 2, each specified by separate structural genes.
CC   -!- SIMILARITY: Belongs to the bacterial flagellin family.
CC   -!- SEQUENCE CAUTION:
CC       Sequence=CAA27129.1; Type=Erroneous initiation;
CC   -----------------------------------------------------------------------
CC   Copyrighted by the UniProt Consortium, see http://www.uniprot.org/terms
CC   Distributed under the Creative Commons Attribution-NoDerivs License
CC   -----------------------------------------------------------------------
DR   EMBL; X03394; CAA27129.1; ALT_INIT; Genomic_DNA.
DR   EMBL; AE017220; AAX65868.1; -; Genomic_DNA.
DR   PIR; S09637; S09637.
DR   RefSeq; YP_216949.1; NC_006905.1.
DR   ProteinModelPortal; P06176; -.
DR   GeneID; 3334444; -.
DR   GenomeReviews; AE017220_GR; SCH_1962.
DR   KEGG; sec:SC1962; -.
DR   PATRIC; 32324831; VBISalEnt136302_2400.
DR   eggNOG; COG1344; -.
DR   KO; K02406; -.
DR   OMA; NSTEDRE; -.
DR   ProtClustDB; PRK08026; -.
DR   BioCyc; SENT321314:SCH_1962-MONOMER; -.
DR   GO; GO:0009420; C:bacterial-type flagellum filament; IEA:InterPro.
DR   GO; GO:0005576; C:extracellular region; IEA:UniProtKB-SubCell.
DR   GO; GO:0005198; F:structural molecule activity; IEA:InterPro.
DR   GO; GO:0001539; P:ciliary or flagellar motility; IEA:InterPro.
DR   Gene3D; G3DSA:2.30.220.10; G3DSA:2.30.220.10; 1.
DR   InterPro; IPR001492; Flagellin.
DR   InterPro; IPR001029; Flagellin_D0/D1.
DR   InterPro; IPR014981; Flagellin_D3.
DR   InterPro; IPR023357; Flagellin_f41_beta-roll_dom.
DR   Pfam; PF00700; Flagellin_C; 1.
DR   Pfam; PF08884; Flagellin_D3; 1.
DR   Pfam; PF00669; Flagellin_N; 1.
DR   PRINTS; PR00207; FLAGELLIN.
PE   3: Inferred from homology;
KW   Bacterial flagellum; Complete proteome; Secreted.
FT   INIT_MET      1      1       Removed (By similarity).
FT   CHAIN         2    501       Flagellin.
FT                                /FTId=PRO_0000182565.
FT   CONFLICT    265    265       T -> R (in Ref. 1; CAA27129).
FT   CONFLICT    296    307       Missing (in Ref. 1).
FT   CONFLICT    348    348       A -> R (in Ref. 1; CAA27129).
FT   CONFLICT    406    406       A -> R (in Ref. 1; CAA27129).
SQ   SEQUENCE   501 AA;  52112 MW;  69F85666ADD866D3 CRC64;
     MAQVINTNSL SLLTQNNLNK SQSALGTAIE RLSSGLRINS AKDDAAGQAI ANRFTANIKG
     LTQASRNAND GISIAQTTEG ALNEINNNLQ RVRELAVQSA NSTNSQSDLD SIQAEITQRL
     NEIDRVSGQT QFNGVKVLAQ DNTLTIQVGA NDGETIDIDL KQINSQTLGL DTLNVQKKYD
     VSDTAVAASY SDSKQNIAVP DKTAITAKIG AATSGGAGIK ADISFKDGKY YATVSGYDDA
     ADTDKNGTYE VTVAADTGAV TFATTPTVVD LPTDAKAVSK VQQNDTEIAA TNAKAALKAA
     GVADAEADTA TLVKMSYTDN NGKVIDGGFA FKTSGGYYAA SVDKSGAASL KVTSYVDATT
     GTEKTAANKL GGADGKTEVV TIDGKTYNAS KAAGHNFKAQ PELAEAAATT TENPLQKIDA
     ALAQVDALRS DLGAVQNRFN SAITNLGNTV NNLSSARSRI EDSDYATEVS NMSRAQILQQ
     AGTSVLAQAN QVPQNVLSLL R
```
