# Supplementary material for: Relationship of Triamine-Biocide Tolerance of Salmonella enterica Serovar Senftenberg to Antimicrobial Susceptibility, Serum Resistance and Outer Membrane Proteins
Source: Int J Mol Sci. 2017 Jul 11;18(7):1459. doi: 10.3390/ijms18071459 (PMC5535950; doi:10.3390/ijms18071459)
Supplement: Supplementary file 1 [file ijms-18-01459-s001.zip › ijms-205418-supplementary/S4_File.pdf]

Mascot Search Results

Protein View

Match to: **ENO_SALAR** Score: **61**
Enolase OS=Salmonella arizonae (strain ATCC BAA-731 / CDC346-86 / RSK2980) GN=eno PE=3 SV=1
Found in search of 281474976711718.mgf

Nominal mass (M_r): **45628**; Calculated pI value: **5.25**
NCBI BLAST search of ENO_SALAR against nr
Unformatted sequence string for pasting into other applications

Taxonomy: Salmonella enterica subsp. arizonae serovar 62:z4,z23:--

Fixed modifications: Carboxymethyl (C)
Variable modifications: Oxidation (M)
Cleavage by Trypsin: cuts C-term side of KR unless next residue is P
Sequence Coverage: **3%**

Matched peptides shown in **Bold Red**

1 MSKIVKVIGR EIIDSRGNPT VEAEVHLEGG FVGMAAAPSG ASTGSREALE
51 LRDGDKSRFL GKGVTKAVGA VNGPIAQAIL GKDAKDQAGI DKIMIDLDGT
101 ENKSNFGANA ILAVSLANAK AAAAAKGMPL YEHIAELNGT PGKYSMPVPM
151 MNIINGGEHA DNNVDIQEFM IQPVGAKTVK EAIRMGSEVF HHLAKVLKGK
201 GMNTAVGDEG GYAPNLGSNA EALAVIAEAV KAAGYELGKD ITLAMDCAAS
251 EFYKDGKYVL AGEGNK**AFTS** **EEFTHFLEEL** **TK**QYPIVSIE DGLDESDWDG
301 FAYQTKVLGD KIQLVGDDLF VTNTKILKEG IEKGIANSIL IKFNQIGSLT
351 ETLAAIKMAK DAGYTAVISH RSGETEDATI ADLAVGTAAG QIKTGSMSRS
401 DRVAKYNQLI RIEEALGEKA PYNGRKEIKG QA

Show predicted peptides also

Sort Peptides By ◉ Residue Number ○ Increasing Mass ○ Decreasing Mass

Start - End	Observed	Mr(expt)	Mr(calc)	ppm	Miss	Sequence	
267 - 282	1928.8306	1927.8233	1927.9203	-50	0	K.AFTSEEFTHFLEELTK.Q	(Ions score 61)

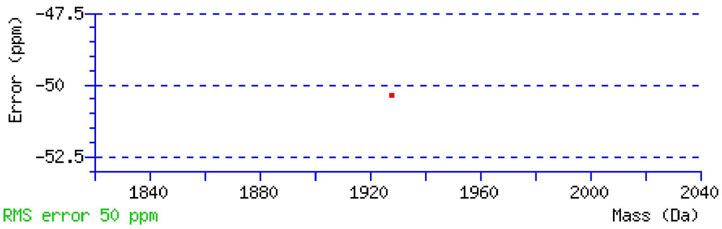

ID   ENO_SALAR   Reviewed;   432 AA.
AC   A9MF11;
DT   20-MAY-2008, integrated into UniProtKB/Swiss-Prot.
DT   05-FEB-2008, sequence version 1.
DT   16-MAY-2012, entry version 31.
DE   RecName: Full=Enolase;
DE   EC=4.2.1.11;
DE   AltName: Full=2-phospho-D-glycerate hydro-lyase;
DE   AltName: Full=2-phosphoglycerate dehydratase;
GN   Name=eno; OrderedLocusNames=SARI_00009;
OS   Salmonella arizonae (strain ATCC BAA-731 / CDC346-86 / RSK2980).
OC   Bacteria; Proteobacteria; Gammaproteobacteria; Enterobacteriales;
OC   Enterobacteriaceae; Salmonella.
OX   NCBI_TaxID=41514;
RN   [1]
RP   NUCLEOTIDE SEQUENCE [LARGE SCALE GENOMIC DNA].
RC   STRAIN=ATCC BAA-731 / CDC346-86 / RSK2980;
RG   The Salmonella enterica serovar Arizonae Genome Sequencing Project;
RA   McClelland M., Sanderson E.K., Porwollik S., Spieth J., Clifton W.S.,
RA   Fulton R., Chunyan W., Wollam A., Shah N., Pepin K., Bhonagiri V.,
RA   Nash W., Johnson M., Thiruvilangam P., Wilson R.;
RL   Submitted (NOV-2007) to the EMBL/GenBank/DDBJ databases.
CC   -!- FUNCTION: Catalyzes the reversible conversion of 2-
CC   phosphoglycerate into phosphoenolpyruvate. It is essential for the
CC   degradation of carbohydrates via glycolysis. It is also a

```
CC       component of the RNA degradosome, a multi-enzyme complex involved
CC       in RNA processing and messenger RNA degradation. Its interaction
CC       with RNase E is important for the turnover of mRNA, in particular
CC       on transcripts encoding enzymes of energy-generating metabolic
CC       routes. Its presence in the degradosome is required for the
CC       response to excess phosphosugar. May play a regulatory role in the
CC       degradation of specific RNAs, such as ptsG mRNA, therefore linking
CC       cellular metabolic status with post-translational gene regulation
CC       (By similarity).
CC   -!- CATALYTIC ACTIVITY: 2-phospho-D-glycerate = phosphoenolpyruvate +
CC       H(2)O.
CC   -!- COFACTOR: Magnesium. Required for catalysis and for stabilizing
CC       the dimer (By similarity).
CC   -!- ENZYME REGULATION: The covalent binding to the substrate causes
CC       inactivation of the enzyme, and possibly serves as a signal for
CC       the export of the protein (By similarity).
CC   -!- PATHWAY: Carbohydrate degradation; glycolysis; pyruvate from D-
CC       glyceraldehyde 3-phosphate: step 4/5.
CC   -!- SUBUNIT: Homodimer. Interacts with the C-terminal region of the
CC       endoribonuclease RNase E in the RNA degradosome (By similarity).
CC   -!- SUBCELLULAR LOCATION: Cytoplasm, cytoskeleton. Secreted. Cell
CC       surface. Note=Fractions of enolase are present in both the
CC       cytoplasm and on the cell surface. As part of the bacterial
CC       cytoskeleton in the cytoplasm, is organized as extended coiled
CC       structures that wind around the cell, from one cell pole to the
CC       other. The export of enolase possibly depends on the covalent
CC       binding to the substrate; once secreted, it remains attached to
CC       the bacterial cell surface (By similarity).
CC   -!- SIMILARITY: Belongs to the enolase family.
```

```
CC   -----------------------------------------------------------------------
CC   Copyrighted by the UniProt Consortium, see http://www.uniprot.org/terms
CC   Distributed under the Creative Commons Attribution-NoDerivs License
CC   -----------------------------------------------------------------------
```

```
DR   EMBL; CP000880; ABX19963.1; -; Genomic_DNA.
DR   RefSeq; YP_001569105.1; NC_010067.1.
DR   ProteinModelPortal; A9MF11; -.
DR   SMR; A9MF11; 2-432.
DR   STRING; A9MF11; -.
DR   PRIDE; A9MF11; -.
DR   GeneID; 5760517; -.
DR   GenomeReviews; CP000880_GR; SARI_00009.
DR   KEGG; ses:SARI_00009; -.
DR   PATRIC; 18469205; VBISalEnt13497_0010.
DR   eggNOG; COG0148; -.
DR   HOGENOM; HOG000072174; -.
DR   KO; K01689; -.
DR   OMA; QEYMIMP; -.
DR   ProtClustDB; PRK00077; -.
DR   GO; GO:0009986; C:cell surface; IEA:UniProtKB-SubCell.
DR   GO; GO:0005856; C:cytoskeleton; IEA:UniProtKB-SubCell.
DR   GO; GO:0005576; C:extracellular region; IEA:UniProtKB-SubCell.
DR   GO; GO:0000015; C:phosphopyruvate hydratase complex; IEA:InterPro.
DR   GO; GO:0000287; F:magnesium ion binding; IEA:InterPro.
DR   GO; GO:0004634; F:phosphopyruvate hydratase activity; IEA:EC.
DR   GO; GO:0006096; P:glycolysis; IEA:UniProtKB-KW.
DR   HAMAP; MF_00318; Enolase; 1; -.
DR   InterPro; IPR000941; Enolase.
DR   InterPro; IPR020810; Enolase_C.
DR   InterPro; IPR020809; Enolase_CS.
DR   InterPro; IPR020811; Enolase_N.
DR   PANTHER; PTHR11902; Enolase; 1.
DR   Pfam; PF00113; Enolase_C; 1.
DR   Pfam; PF03952; Enolase_N; 1.
DR   PIRSF; PIRSF001400; Enolase; 1.
DR   PRINTS; PR00148; ENOLASE.
DR   TIGRFAMs; TIGR01060; Eno; 1.
DR   PROSITE; PS00164; ENOLASE; 1.
PE   3: Inferred from homology;
KW   Complete proteome; Cytoplasm; Cytoskeleton; Glycolysis; Lyase;
KW   Magnesium; Metal-binding; Phosphoprotein; Secreted.
FT   CHAIN         1    432       Enolase.
FT                                /FTId=PRO_1000079147.
FT   REGION      369    372       Substrate binding (By similarity).
FT   ACT_SITE    209    209       Proton donor (By similarity).
FT   ACT_SITE    342    342       Proton acceptor (By similarity).
FT   METAL       246    246       Magnesium (By similarity).
FT   METAL       290    290       Magnesium (By similarity).
FT   METAL       317    317       Magnesium (By similarity).
FT   BINDING     159    159       Substrate (By similarity).
FT   BINDING     168    168       Substrate (By similarity).
```

```
FT   BINDING     290    290       Substrate (By similarity).
FT   BINDING     317    317       Substrate (By similarity).
FT   BINDING     342    342       Substrate (covalent); in inhibited form
FT                                (By similarity).
FT   BINDING     393    393       Substrate (By similarity).
FT   MOD_RES     284    284       Phosphotyrosine (By similarity).
SQ   SEQUENCE   432 AA;  45599 MW;  2A9984B6784DCD08 CRC64;
     MSKIVKVIGR EIIDSRGNPT VEAEVHLEGG FVGMAAAPSG ASTGSREALE LRDGDKSRFL
     GKGVTKAVGA VNGPIAQAIL GKDAKDQAGI DKIMIDLDGT ENKSNFGANA ILAVSLANAK
     AAAAAKGMPL YEHIAELNGT PGKYSMPVPM MNIINGGEHA DNNVDIQEFM IQPVGAKTVK
     EAIRMGSEVF HHLAKVLKGK GMNTAVGDEG GYAPNLGSNA EALAVIAEAV KAAGYELGKD
     ITLAMDCAAS EFYKDGKYVL AGEGNKAFTS EEFTHFLEEL TKQYPIVSIE DGLDESDWDG
     FAYQTKVLGD KIQLVGDDLF VTNTKILKEG IEKGIANSIL IKFNQIGSLT ETLAAIKMAK
     DAGYTAVISH RSGETEDATI ADLAVGTAAG QIKTGSMSRS DRVAKYNQLI RIEEALGEKA
     PYNGRKEIKG QA
```

Mascot: http://www.matrixscience.com/
