# Supplementary material for: Relationship of Triamine-Biocide Tolerance of Salmonella enterica Serovar Senftenberg to Antimicrobial Susceptibility, Serum Resistance and Outer Membrane Proteins
Source: Int J Mol Sci. 2017 Jul 11;18(7):1459. doi: 10.3390/ijms18071459 (PMC5535950; doi:10.3390/ijms18071459)
Supplement: Supplementary file 1 [file ijms-18-01459-s001.zip › ijms-205418-supplementary/S5_File.pdf]

Mascot Search Results

Protein View

Match to: **CHEB_SALCH** Score: **30**
Chemotaxis response regulator protein-glutamate methylesterase OS=Salmonella choleraesuis (strain SC-B67) GN=cheB PE=3 SV=1
Found in search of 281474976711693.mgf

Nominal mass (M_r): **37498**; Calculated pI value: **8.48**
NCBI BLAST search of CHEB_SALCH against nr
Unformatted sequence string for pasting into other applications

Taxonomy: Salmonella enterica subsp. enterica serovar Choleraesuis str. SC-B67

Variable modifications: Carboxymethyl (C),Oxidation (M)
Cleavage by Trypsin: cuts C-term side of KR unless next residue is P
Sequence Coverage: **2%**

Matched peptides shown in **Bold Red**

```
  1 MSKIRVLSVD DSALMRQIMT EIINSHSDME MVATAPDPLV ARDLIKKFNP
 51 DVLTLDVEMP RMDGLDFLEK LMRLRPMPVV MVSSLTGKGS EVTLRALELG
101 AIDFVTKPQL GIREGMLAYS EMIAEKVRTA ARARIAAHKP MAAPATLKAG
151 PLLSSEKLIA IGASTGGTEA IRHVLQPLPL SSPAVIITQH MPPGFTRSFA
201 ERLNKLCQIS VKEAEDGERV LPGHAYIAPG DKHMELARSG ANYQIKIHDG
251 PPVNRHRPSV DVLFHSVAKH AGRNAVGVIL TGMGNDGAAG MLAMYQAGAW
301 TIAQNEASCV VFGMPREAIN MGGVSEVVDL SQVSQQMLAK ISAGQAIRI
```

Show predicted peptides also

Sort Peptides By ◉ Residue Number ○ Increasing Mass ○ Decreasing Mass

Start - End	Observed	Mr(expt)	Mr(calc)	ppm	Miss	Sequence	
341 - 349	928.5114	927.5041	927.5501	-50	1	K.ISAGQAIRI.-	(Ions score 30)

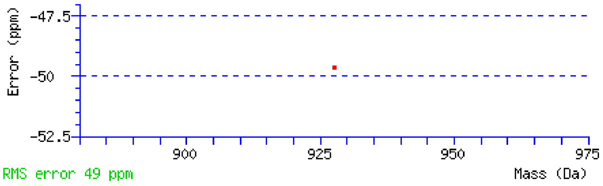


```
ID   CHEB_SALCH              Reviewed;         349 AA.
AC   Q57N81;
DT   07-MAR-2006, integrated into UniProtKB/Swiss-Prot.
DT   10-MAY-2005, sequence version 1.
DT   16-MAY-2012, entry version 56.
DE   RecName: Full=Chemotaxis response regulator protein-glutamate methylesterase;
DE            EC=3.1.1.61;
GN   Name=cheB; OrderedLocusNames=SCH_1924;
OS   Salmonella choleraesuis (strain SC-B67).
OC   Bacteria; Proteobacteria; Gammaproteobacteria; Enterobacteriales;
OC   Enterobacteriaceae; Salmonella;
OC   Salmonella enterica subsp. enterica serovar Choleraesuis.
OX   NCBI_TaxID=321314;
RN   [1]
RP   NUCLEOTIDE SEQUENCE [LARGE SCALE GENOMIC DNA].
RC   STRAIN=SC-B67;
RX   PubMed=15781495; DOI=10.1093/nar/gki297;
RA   Chiu C.-H., Tang P., Chu C., Hu S., Bao Q., Yu J., Chou Y.-Y.,
RA   Wang H.-S., Lee Y.-S.;
RT   "The genome sequence of Salmonella enterica serovar Choleraesuis, a
RT   highly invasive and resistant zoonotic pathogen.";
RL   Nucleic Acids Res. 33:1690-1698(2005).
CC   -!- FUNCTION: Involved in the modulation of the chemotaxis system;
CC       catalyzes the demethylation of specific methylglutamate residues
CC       introduced into the chemoreceptors (methyl-accepting chemotaxis
CC       proteins) by CheR (By similarity).
CC   -!- CATALYTIC ACTIVITY: Protein L-glutamate O(5)-methyl ester + H(2)O
CC       = protein L-glutamate + methanol.
CC   -!- SUBCELLULAR LOCATION: Cytoplasm.
CC   -!- DOMAIN: The N-terminal regulatory domain inhibits the activity of
CC       the C-terminal effector domain.
CC   -!- PTM: Phosphorylated by CheA. Phosphorylation suppresses the
CC       inhibitory activity of the N-terminal domain (By similarity).
CC   -!- SIMILARITY: Contains 1 cheB-type methylesterase domain.
CC   -!- SIMILARITY: Contains 1 response regulatory domain.
CC   -----------------------------------------------------------------------
CC   Copyrighted by the UniProt Consortium, see http://www.uniprot.org/terms
CC   Distributed under the Creative Commons Attribution-NoDerivs License
CC   -----------------------------------------------------------------------
DR   EMBL; AE017220; AAX65830.1; -; Genomic_DNA.
DR   RefSeq; YP_216911.1; NC_006905.1.
DR   ProteinModelPortal; Q57N81; -.
DR   SMR; Q57N81; 1-349.
```

```
DR   GeneID; 3334405; -.
DR   GenomeReviews; AE017220_GR; SCH_1924.
DR   KEGG; sec:SC1924; -.
DR   PATRIC; 32324739; VBISalEnt136302_2357.
DR   eggNOG; COG2201; -.
DR   KO; K03412; -.
DR   OMA; FVTKPKL; -.
DR   ProtClustDB; PRK00742; -.
DR   BioCyc; SENT321314:SCH_1924-MONOMER; -.
DR   GO; GO:0005737; C:cytoplasm; IEA:UniProtKB-SubCell.
DR   GO; GO:0008984; F:protein-glutamate methylesterase activity; IEA:EC.
DR   GO; GO:0000156; F:two-component response regulator activity; IEA:InterPro.
DR   GO; GO:0006935; P:chemotaxis; IEA:UniProtKB-KW.
DR   GO; GO:0006355; P:regulation of transcription, DNA-dependent; IEA:InterPro.
DR   Gene3D; G3DSA:3.40.50.180; Chemotax_RR_pGlu_Me-esterase; 1.
DR   HAMAP; MF_00099; CheB_methylest; 1; -.
DR   InterPro; IPR011006; CheY-like_superfamily.
DR   InterPro; IPR008248; Sig_transdc_resp-reg_CheB.
DR   InterPro; IPR000673; Sig_transdc_resp-reg_Me-estase.
DR   InterPro; IPR001789; Sig_transdc_resp-reg_receiver.
DR   Pfam; PF01339; CheB_methylest; 1.
DR   Pfam; PF00072; Response_reg; 1.
DR   PIRSF; PIRSF000876; RR_chemtxs_CheB; 1.
DR   SMART; SM00448; REC; 1.
DR   SUPFAM; SSF52738; Chemotax_RR_pGlu_Me-esterase; 1.
DR   SUPFAM; SSF52172; CheY_like; 1.
DR   PROSITE; PS50122; CHEB; 1.
DR   PROSITE; PS50110; RESPONSE_REGULATORY; 1.
PE   3: Inferred from homology;
KW   Chemotaxis; Complete proteome; Cytoplasm; Hydrolase; Phosphoprotein.
FT   CHAIN         1    349       Chemotaxis response regulator protein-
FT                                glutamate methylesterase.
FT                                /FTId=PRO_0000225483.
FT   DOMAIN        5    122       Response regulatory.
FT   DOMAIN      152    344       CheB-type methylesterase.
FT   ACT_SITE    164    164       By similarity.
FT   ACT_SITE    190    190       By similarity.
FT   ACT_SITE    286    286       By similarity.
FT   MOD_RES      56     56       4-aspartylphosphate (By similarity).
SQ   SEQUENCE   349 AA;  37522 MW;  82B408862A8F9590 CRC64;
     MSKIRVLSVD DSALMRQIMT EIINSHSDME MVATAPDPLV ARDLIKKFNP DVLTLDVEMP
     RMDGLDFLEK LMRLRPMPVV MVSSLTGKGS EVTLRALELG AIDFVTKPQL GIREGMLAYS
     EMIAEKVRTA ARARIAAHKP MAAPATLKAG PLLSSEKLIA IGASTGGTEA IRHVLQPLPL
     SSPAVIITQH MPPGFTRSFA ERLNKLCQIS VKEAEDGERV LPGHAYIAPG DKHMELARSG
     ANYQIKIHDG PPVNRHRPSV DVLFHSVAKH AGRNAVGVIL TGMGNDGAAG MLAMYQAGAW
     TIAQNEASCV VFGMPREAIN MGGVSEVVDL SQVSQQMLAK ISAGQAIRI
```

Mascot: http://www.matrixscience.com/
