# Supplementary material for: Relationship of Triamine-Biocide Tolerance of Salmonella enterica Serovar Senftenberg to Antimicrobial Susceptibility, Serum Resistance and Outer Membrane Proteins
Source: Int J Mol Sci. 2017 Jul 11;18(7):1459. doi: 10.3390/ijms18071459 (PMC5535950; doi:10.3390/ijms18071459)
Supplement: Supplementary file 1 [file ijms-18-01459-s001.zip › ijms-205418-supplementary/S6_File.pdf]

Mascot Search Results

Protein View

Match to: **BAMA_SALTI** Score: **67** Expect: **0.0025**
Outer membrane protein assembly factor BamA OS=Salmonella typhi GN=bamA PE=3 SV=1

Nominal mass (M_r): **89525**; Calculated pI value: **4.92**
NCBI BLAST search of BAMA_SALTI against nr
Unformatted sequence string for pasting into other applications

Taxonomy: Salmonella enterica subsp. enterica serovar Typhi

Fixed modifications: Carbamidomethyl (C)
Variable modifications: Oxidation (M)
Cleavage by Trypsin: cuts C-term side of KR unless next residue is P
Number of mass values searched: **50**
Number of mass values matched: **10**
Sequence Coverage: **19%**

Matched peptides shown in **Bold Red**

1 MAMKKLLIAS LLFSSATVYG AEGFVVKDIH FEGLQRVAVG AALLSMPVRT
51 GDTVNDEDIS NTIR**ALFATG NFEDVRVLR**D GNTLLVQVKE RPTIASITFS
101 GNKSVKDDML K**QNLEASGVR** VGESLDRTTL SDIEK**GLEDF YYSVGK**YSAS
151 VKAVVTPLPR NR**VDLKLVFQ EGVSAK**IQQI NIVGNHAFST EELISHFQLR
201 DEVPWWNVVG DRKYQKQKLA GDLETLR**SYY LDRGYAR**FNI DSTQVSLTPD
251 KKGIYITVNI TEGDQYK**LSG VQVSGNLAGH SAEIEK**LTKI EPGELYNGTK
301 VTKMEDDIKK LLGRYGYAYP RVQSQPEIND ADKTVKLRVN VDAGNRFYVR
351 KIRFEGNDTS KDSVLRR**EMR QMEGAWLGSD LVDQGK**ERLN RLGFFETVDT
401 DTQRVPGSPD QVDVVYKVKE RNTGSFNFGI GYGTESGVSF QAGVQQDNWL
451 GTGYSVGING TKNDYQTYSE LSVTNPYFTV DGVSLGGRIF YNDFEADDAD
501 LSDYTNKSYG TDVTLGFPIN EYNTLR**AGLG YVHNKLSNMQ PQIAMDR**YLE
551 SMGDPDASDF AADDFTFNYG WTYNK**LDRGY FPTDGSR**VNL TGKVTIPGSD
601 NEYYKVSLDT ATYVPIDNDH KWVVLGRTRW GYGDGLGGKE MPFYENFYAG
651 GSSTVRGFQS NTIGPKAVYK **NGAHTSWDDD DDYEDCTQES GCK**SDDAVGG
701 NAMAVASLEF ITPTPFISEK YANSVRTSFF WDMGTVWDTN WDPSSAPSDV
751 PDYSDPGNIR MSAGIALQWM SPLGPLVFSY AQPFKKYDGD KAEQFQFNIG
801 KTW

Show predicted peptides also

Sort Peptides By ◉ Residue Number ○ Increasing Mass ○ Decreasing Mass

Start - End	Observed	Mr(expt)	Mr(calc)	ppm	Miss	Sequence
65 - 79	1707.7742	1706.7670	1706.9104	-84	1	R.ALFATGNFEDVRVLR.D
112 - 120	973.5347	972.5274	972.4988	29	0	K.QNLEASGVR.V
136 - 146	1277.7129	1276.7056	1276.5976	85	0	K.GLEDFYYSVGK.Y
163 - 176	1532.7870	1531.7797	1531.8610	-53	1	R.VDLKLVFQEGVSAK.I
228 - 237	1263.6956	1262.6883	1262.6044	67	1	R.SYYLDRGYAR.F
268 - 286	1895.9570	1894.9497	1894.9748	-13	0	K.LSGVQVSGNLAGHSAEIEK.L
368 - 386	2150.0771	2149.0698	2148.9932	36	1	R.EMRQMEGAWLGSDLVDQGK.E
527 - 547	2342.9698	2341.9625	2342.1623	-85	1	R.AGLGYVHNKLSNMQPQIAMDR.Y
576 - 587	1383.6925	1382.6852	1382.6579	20	1	K.LDRGYFPTDGSR.V
671 - 693	2705.1059	2704.0986	2703.9562	53	0	K.NGAHTSWDDDDDYEDCTQESGCK.S

No match to: 601.4593, 856.5298, 870.5436, 1107.5504, 1128.5691, 1140.5835, 1157.5879, 1193.6068, 1219.6164, 1233.6807, 1235.6313, 1259.6806,

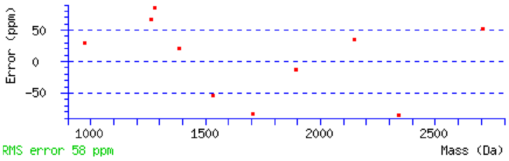

ID   BAMA_SALTI   Reviewed;   803 AA.
AC   Q8Z9A3; Q7CBP9;
DT   20-DEC-2005, integrated into UniProtKB/Swiss-Prot.
DT   01-MAR-2002, sequence version 1.
DT   13-JUN-2012, entry version 59.
DE   RecName: Full=Outer membrane protein assembly factor BamA;
DE   Flags: Precursor;
GN   Name=bamA; Synonyms=yaeT; OrderedLocusNames=STY0247, t0225;
OS   Salmonella typhi.
OC   Bacteria; Proteobacteria; Gammaproteobacteria; Enterobacteriales;
OC   Enterobacteriaceae; Salmonella.
OX   NCBI_TaxID=90370;
RN   [1]
RP   NUCLEOTIDE SEQUENCE [LARGE SCALE GENOMIC DNA].
RC   STRAIN=CT18;
RX   MEDLINE=21534947; PubMed=11677608; DOI=10.1038/35101607;
RA   Parkhill J., Dougan G., James K.D., Thomson N.R., Pickard D., Wain J.,
RA   Churcher C.M., Mungall K.L., Bentley S.D., Holden M.T.G., Sebaihia M.,
RA   Baker S., Basham D., Brooks K., Chillingworth T., Connerton P.,
RA   Cronin A., Davis P., Davies R.M., Dowd L., White N., Farrar J.,
RA   Feltwell T., Hamlin N., Haque A., Hien T.T., Holroyd S., Jagels K.,
RA   Krogh A., Larsen T.S., Leather S., Moule S., O'Gaora P., Parry C.,
RA   Quail M.A., Rutherford K.M., Simmonds M., Skelton J., Stevens K.,
RA   Whitehead S., Barrell B.G.;
RT   "Complete genome sequence of a multiple drug resistant Salmonella
RT   enterica serovar Typhi CT18.";
RL   Nature 413:848-852(2001).
RN   [2]
RP   NUCLEOTIDE SEQUENCE [LARGE SCALE GENOMIC DNA].
RC   STRAIN=ATCC 700931 / Ty2;
RX   MEDLINE=22531367; PubMed=12644504;
RX   DOI=10.1128/JB.185.7.2330-2337.2003;
RA   Deng W., Liou S.-R., Plunkett G. III, Mayhew G.F., Rose D.J.,

```
RA   Burland V., Kodoyianni V., Schwartz D.C., Blattner F.R.;
RT   "Comparative genomics of Salmonella enterica serovar Typhi strains Ty2
RT   and CT18.";
RL   J. Bacteriol. 185:2330-2337(2003).
CC   -!- FUNCTION: Part of the outer membrane protein assembly complex,
CC       which is involved in assembly and insertion of beta-barrel
CC       proteins into the outer membrane. Constitutes, with BamD, the core
CC       component of the assembly machinery (By similarity).
CC   -!- SUBUNIT: Part of the Bam complex, which is composed of the outer
CC       membrane protein BamA, and four lipoproteins BamB, BamC, BamD and
CC       BamE (By similarity).
CC   -!- SUBCELLULAR LOCATION: Cell outer membrane (By similarity).
CC   -!- SIMILARITY: Belongs to the BamA family.
CC   -----------------------------------------------------------------------
CC   Copyrighted by the UniProt Consortium, see http://www.uniprot.org/terms
CC   Distributed under the Creative Commons Attribution-NoDerivs License
CC   -----------------------------------------------------------------------
DR   EMBL; AL627266; CAD08682.1; -; Genomic_DNA.
DR   EMBL; AE014613; AAO67955.1; -; Genomic_DNA.
DR   RefSeq; NP_454831.1; NC_003198.1.
DR   RefSeq; NP_804106.1; NC_004631.1.
DR   ProteinModelPortal; Q8Z9A3; -.
DR   SMR; Q8Z9A3; 21-351.
DR   GeneID; 1067818; -.
DR   GeneID; 1246736; -.
DR   GenomeReviews; AE014613_GR; t0225.
DR   GenomeReviews; AL513382_GR; STY0247.
DR   KEGG; stt:t0225; -.
DR   KEGG; sty:STY0247; -.
DR   PATRIC; 18538311; VBISalEnt120419_0247.
DR   eggNOG; COG4775; -.
DR   HOGENOM; HOG000261766; -.
DR   KO; K07277; -.
DR   OMA; NSKTRDE; -.
DR   ProtClustDB; PRK11067; -.
DR   BioCyc; SENT209261:T0225-MONOMER; -.
DR   BioCyc; SENT220341:STY0247-MONOMER; -.
DR   GO; GO:0009279; C:cell outer membrane; IEA:UniProtKB-SubCell.
DR   GO; GO:0016021; C:integral to membrane; IEA:UniProtKB-KW.
DR   GO; GO:0005886; C:plasma membrane; IEA:UniProtKB-KW.
DR   HAMAP; MF_01430; OM_assembly_BamA; 1; -.
DR   InterPro; IPR000184; Bac_surfAg_D15.
DR   InterPro; IPR023707; OM_YaeT/YfiO_complex_suYaeT.
DR   InterPro; IPR016474; OM_YaeT/YfiO_complex_YaeT-rel.
DR   InterPro; IPR010827; Surface_Ag_variable_number.
DR   Pfam; PF01103; Bac_surface_Ag; 1.
DR   Pfam; PF07244; Surf_Ag_VNR; 5.
DR   PIRSF; PIRSF006076; OM_assembly_OMP85; 1.
DR   TIGRFAMs; TIGR03303; OM_YaeT; 1.
PE   3: Inferred from homology;
KW   Cell membrane; Cell outer membrane; Complete proteome; Membrane;
KW   Signal; Transmembrane; Transmembrane beta strand.
FT   SIGNAL        1     20       Potential.
FT   CHAIN        21    803       Outer membrane protein assembly factor
FT                                BamA.
FT                                /FTId=PRO_0000045376.
SQ   SEQUENCE   803 AA;  89467 MW;  BBF8670070E67628 CRC64;
     MAMKKLLIAS LLFSSATVYG AEGFVVKDIH FEGLQRVAVG AALLSMPVRT GDTVNDEDIS
     NTIRALFATG NFEDVRVLRD GNTLLVQVKE RPTIASITFS GNKSVKDDML KQNLEASGVR
     VGESLDRTTL SDIEKGLEDF YYSVGKYSAS VKAVVTPLPR NRVDLKLVFQ EGVSAKIQQI
     NIVGNHAFST EELISHFQLR DEVPWWNVVG DRKYQKQKLA GDLETLRSYY LDRGYARFNI
     DSTQVSLTPD KKGIYITVNI TEGDQYKLSG VQVSGNLAGH SAEIEKLTKI EPGELYNGTK
     VTKMEDDIKK LLGRYGYAYP RVQSQPEIND ADKTVKLRVN VDAGNRFYVR KIRFEGNDTS
     KDSVLRREMR QMEGAWLGSD LVDQGKERLN RLGFFETVDT DTQRVPGSPD QVDVVYKVKE
     RNTGSFNFGI GYGTESGVSF QAGVQQDNWL GTGYSVGING TKNDYQTYSE LSVTNPYFTV
     DGVSLGGRIF YNDFEADDAD LSDYTNKSYG TDVTLGFPIN EYNTLRAGLG YVHNKLSNMQ
     PQIAMDRYLE SMGDPDASDF AADDFTFNYG WTYNKLDRGY FPTDGSRVNL TGKVTIPGSD
     NEYYKVSLDT ATYVPIDNDH KWVVLGRTRW GYGDGLGGKE MPFYENFYAG GSSTVRGFQS
     NTIGPKAVYK NGAHTSWDDD DDYEDCTQES GCKSDDAVGG NAMAVASLEF ITPTPFISEK
     YANSVRTSFF WDMGTVWDTN WDPSSAPSDV PDYSDPGNIR MSAGIALQWM SPLGPLVFSY
     AQPFKKYDGD KAEQFQFNIG KTW
```

Mascot: http://www.matrixscience.com/
