# Supplementary material for: Relationship of Triamine-Biocide Tolerance of Salmonella enterica Serovar Senftenberg to Antimicrobial Susceptibility, Serum Resistance and Outer Membrane Proteins
Source: Int J Mol Sci. 2017 Jul 11;18(7):1459. doi: 10.3390/ijms18071459 (PMC5535950; doi:10.3390/ijms18071459)
Supplement: Supplementary file 1 [file ijms-18-01459-s001.zip › ijms-205418-supplementary/S1_Table.doc]

**Table S1.** Bactericidal activity of 22.5% NHS and 22.5% HIS decomplemented by heating at 56°C for 30 min against *S.* Senftenberg wild-type strains and their biocide-resistant variants.

| **Strain** |  | | **Bactericidal activity of 22.5% NHS** | | | | | | **Bactericidal activity of 22.5% HIS** | | | | | |
| --- | --- | --- | --- | --- | --- | --- | --- | --- | --- | --- | --- | --- | --- | --- |
|  | **CFU/ml** | | | | **Survival rate of cells at T_1_ [%]** | **RP** | **Survival rate of cells at T_2_ [%]** | **RP** | **CFU/ml** | | | **Survival rate of cells at T_1_ [%]** | **Survival rate of cells at T_2_ [%]** | **RP** |
|  | **T_0_** | **T_1_** | | **T_2_** |  |  |  |  | **T_0_** | **T_1_** | **T_2_** |  |  |  |
| 131 | 3.3 × 10^5^ ±4.7 × 10^4^ | 1.7 × 10^5^ ±4.7 × 10^4^ | | 1.3 × 10^4^ ±3.8 × 10^3^ | 50.0 | **R** | 3.8 | S | 3.2 × 10^5^ ±1.5 × 10^5^ | 3.7 × 10^5^ ±4.7 × 10^4^ | 3.3 × 10^5^ ±4.7 × 10^4^ | 115.8 | 105.3 | R |
| 131^bST^ | 1.7 × 10^5^ ±4.7 × 10^4^ | 2.5 × 10^5^ ±1.0 × 10^5^ | | 1.2 × 10^4^ ±2.4 × 10^3^ | 152.0 | **R** | 7.0 | S | 1.7 × 10^5^ ±4.7 × 10^4^ | 3.3 × 10^5^ ±1.9 × 10^5^ | 2.0 × 10^5^ ±8.2 × 10^4^ | 200.0 | 120.0 | R |
| 131^aST^ | 1.7 × 10^5^ ±9.4 × 10^4^ | 1.2 × 10^5^ ±2.4 × 10^4^ | | 1.3 × 10^5^ ±4.7 × 10^4^ | 70.0 | **R** | 80.0 | **R** | 1.3 × 10^5^ ±4.7 × 10^4^ | 1.3 × 10^5^ ±4.7 × 10^4^ | 1.3 × 10^5^ ±4.7 × 10^4^ | 100.0 | 100.0 | R |
| 132 | 3.3 × 10^5^ ±1.2 × 10^5^ | 6.0 × 10^5^ ±3.6 × 10^5^ | | 1.2 × 10^4^ ±2.4 × 10^3^ | 180.0 | **R** | 3.5 | S | 2.3 × 10^5^ ±9.4 × 10^4^ | 2.3 × 10^5^ ±4.7 × 10^4^ | 2.7 × 10^5^ ±1.2 × 10^5^ | 100.0 | 114.3 | R |
| 132^bST^ | 2.7 × 10^5^ ±4.7 × 10^4^ | 1.2 × 10^5^ ±2.8 × 10^4^ | | 3.7 × 10^5^ ±1.2 × 10^5^ | 45.0 | S | 137.5 | **R** | 3.9 × 10^5^ ±1.3 × 10^5^ | 4.0 × 10^5^ ±8.2 × 10^4^ | 7.7 × 10^5^ ±4.7 × 10^4^ | 103.4 | 198.3 | R |
| 132^aST^ | 1.1 × 10^5^ ±1.9 × 10^4^ | 1.9 × 10^5^ ±2.1 × 10^4^ | | 1.1 × 10^4^ ±1.9 × 10^3^ | 170.6 | **R** | 10.0 | S | 1.3 × 10^5^ ±4.7 × 10^4^ | 1.5 × 10^5^ ±4.1 × 10^4^ | 1.7 × 10^5^ ±4.7 × 10^4^ | 115.0 | 125.0 | R |
| 133 | 1.3 × 10^5^ ±4.7 × 10^4^ | 2.0 × 10^4^ ±8.2 × 10^3^ | | 5.4 × 10^1^ ±3.2 × 10^1^ | 15.0 | S | 0 | S | 3.1 × 10^5^ ±1.6 × 10^5^ | 4.7 × 10^5^ ±2.1 × 10^5^ | 5.7 × 10^5^ ±4.7 × 10^4^ | 152.2 | 184.8 | R |
| 133^bST^ | 4.0 × 10^4^ ±8.2 × 10^3^ | 9.2 × 10^1^ ±1.1 × 10^1^ | | 0 | 0.2 | S | 0 | S | 1.7 × 10^5^ ±9.4 × 10^4^ | 3.5 × 10^5^ ±1.4 × 10^5^ | 2.0 × 10^5^ ±8.2 × 10^4^ | 212.0 | 120.0 | R |
| 133^aST^ | 1.1 × 10^5^ ±1.4 × 10^4^ | 1.1 × 10^5^ ±1.9 × 10^4^ | | 0 | 103.0 | **R** | 0 | S | 1.7 × 10^5^ ±9.4 × 10^4^ | 1.6 × 10^5^ ±4.0 × 10^4^ | 3.0 × 10^5^ ±8.2 × 10^4^ | 98.0 | 180.0 | R |
| 134 | 1.7 × 10^5^ ±9.4 × 10^4^ | 1.7 × 10^5^ ±4.7 × 10^4^ | | 1.2 × 10^4^ ±1.6 × 10^3^ | 100.0 | **R** | 7.2 | S | 1.7 × 10^5^ ±4.7 × 10^4^ | 2.0 × 10^5^ ±8.2 × 10^4^ | 1.7 × 10^5^ ±9.4 × 10^4^ | 120.0 | 100.0 | R |
| 134^bST^ | 2.3 × 10^5^ ±4.7 × 10^4^ | 2.2 × 10^5^ ±9.4 × 10^4^ | | 3.0 × 10^5^ ±8.2 × 10^4^ | 94.3 | **R** | 128.6 | **R** | 3.9 × 10^5^ ±1.5 × 10^5^ | 4.0 × 10^5^ ±2.2 × 10^5^ | 7.3 × 10^5^ ±4.7 × 10^4^ | 101.7 | 188.0 | R |
| 134^aST^ | 1.7 × 10^5^ ±3.8 × 10^4^ | 1.7 × 10^5^ ±9.4 × 10^4^ | | 1.3 × 10^5^ ±4.7 × 10^4^ | 96.2 | **R** | 76.9 | **R** | 1.3 × 10^5^ ±4.7 × 10^4^ | 2.0 × 10^5^ ±8.2 × 10^4^ | 2.1 × 10^5^ ±1.9 × 10^4^ | 150.0 | 160.0 | R |
| 135 | 4.0 × 10^5^ ±8.2 × 10^4^ | 4.0 × 10^5^ ±1.6 × 10^5^ | | 2.0 × 10^5^ ±8.2 × 10^4^ | 100.0 | **R** | 50.0 | **R** | 2.7 × 10^5^ ±1.2 × 10^5^ | 3.0 × 10^5^ ±8.2 × 10^4^ | 3.7 × 10^5^ ±1.2 × 10^5^ | 112.5 | 137.5 | R |
| 135^bST^ | 3.1 × 10^5^ ±1.5 × 10^5^ | 1.4 × 10^5^ ±4.5 × 10^4^ | | 1.2 × 10^4^ ±2.8 × 10^3^ | 44.6 | S | 3.9 | S | 1.1 × 10^5^ ±1.4 × 10^4^ | 1.1 × 10^5^ ±1.9 × 10^4^ | 1.3 × 10^5^ ±4.7 × 10^4^ | 103.0 | 121.2 | R |
| 135^aST^ | 1.3 × 10^6^ ±3.8 × 10^5^ | 1.1 × 10^5^ ±1.9 × 10^4^ | | 9.1 × 10^1^ ±1.3 × 10^1^ | 8.9 | S | 0 | S | 1.2 × 10^5^ ±2.4 × 10^4^ | 1.7 × 10^5^ ±4.7 × 10^4^ | 1.8 × 10^5^ ±5.9 × 10^4^ | 142.9 | 157.1 | R |

Definitions of abbreviations: NHS, normal human serum; HIS, heat-inactivated normal human serum; CFU, colony forming units; RP, resistance pattern; S, sensitive; R, resistant; bST, before the test of stability; aST, after the test of stability.
