# Supplementary material for: Relationship of Triamine-Biocide Tolerance of Salmonella enterica Serovar Senftenberg to Antimicrobial Susceptibility, Serum Resistance and Outer Membrane Proteins
Source: Int J Mol Sci. 2017 Jul 11;18(7):1459. doi: 10.3390/ijms18071459 (PMC5535950; doi:10.3390/ijms18071459)
Supplement: Supplementary file 1 [file ijms-18-01459-s001.zip › ijms-205418-supplementary/S2_Fig.doc]

**Figure S2.** Confirmation of resistant to biocide *Salmonella* variants on Brilliant Green agar medium. (**a**) Before the test of phenotypic stability. A. *S.* Senftenberg 131, B. *S.* Senftenberg 132, C. *S.* Senftenberg 133, D. *S.* Senftenberg 134, E. *S.* Senftenberg 135. (**b**) After the test of stability. A. *S.* Senftenberg 131, B. *S.* Senftenberg 132, C. *S.* Senftenberg 133, D. *S.* Senftenberg 134, E. *S.* Senftenberg 135.

(**a**)

(**b**)
