# Supplementary material for: Relationship of Triamine-Biocide Tolerance of Salmonella enterica Serovar Senftenberg to Antimicrobial Susceptibility, Serum Resistance and Outer Membrane Proteins
Source: Int J Mol Sci. 2017 Jul 11;18(7):1459. doi: 10.3390/ijms18071459 (PMC5535950; doi:10.3390/ijms18071459)
Supplement: Supplementary file 1 [file ijms-18-01459-s001.zip › ijms-205418-supplementary/S2_Table.doc]

**Table S2.** Bactericidal activity of 45% NHS and 45% HIS decomplemented by heating at 56°C for 30 min against *S.* Senftenberg wild-type strains and their biocide-resistant variants.

| **Strain** | **Bactericidal activity of 45% NHS** | | | | | | | **Bactericidal activity of 45% HIS** | | | | | |
| --- | --- | --- | --- | --- | --- | --- | --- | --- | --- | --- | --- | --- | --- |
|  | **CFU/ml** | | | **Survival rate of cells at T_1_ [%]** | **RP** | **Survival rate of cells at T_2_ [%]** | **RP** | **CFU/ml** | | | **Survival rate of cells at T_1_ [%]** | **Survival rate of cells at T_2_ [%]** | **RP** |
|  | T_0_ | T_1_ | T_2_ |  |  |  |  | T_0_ | T_1_ | T_2_ |  |  |  |
| 131 | 1.2 × 10^5^ ±9.4 × 10^3^ | 1.3 × 10^4^ ±4.7 × 10^3^ | 1.2 × 10^3^ ±1.6 × 10^2^ | 10.8 | S | 1.0 | S | 1.3 × 10^5^ ±1.7 × 10^4^ | 1.5 × 10^5^ ±8.2 × 10^3^ | 1.6 × 10^5^ ±3.3 × 10^4^ | 118.4 | 128.9 | R |
| 131^bST^ | 1.2 × 10^5^ ±1.6 × 10^4^ | 6.7 × 10^4^ ±1.2 × 10^4^ | 8.7 × 10^3^ ±2.1 × 10^3^ | 55.6 | **R** | 7.2 | S | 1.3 × 10^5^ ±2.4 × 10^4^ | 1.8 × 10^5^ ±6.0 × 10^4^ | 1.4 × 10^5^ ±2.9 × 10^4^ | 135.9 | 107.7 | R |
| 131^aST^ | 1.1 × 10^5^ ±2.6 × 10^4^ | 9.7 × 10^4^ ±1.2 × 10^4^ | 1.2 × 10^5^ ±4.5 × 10^4^ | 85.3 | **R** | 102.9 | **R** | 1.1 × 10^5^ ±2.5 × 10^4^ | 1.0 × 10^5^ ±2.5 × 10^4^ | 1.3 × 10^5^ ±2.1 × 10^4^ | 96.9 | 118.8 | R |
| 132 | 1.6 × 10^5^  ±8.3 × 10^4^ | 1.1 × 10^5^ ±1.4 × 10^4^ | 7.0 × 10^3^ ±8.2 × 10^2^ | 67.3 | **R** | 4.3 | S | 5.0 × 10^4^ ±2.9 × 10^4^ | 5.6 × 10^4^ ±2.1 × 10^4^ | 5.3 × 10^4^ ±9.4 × 10^3^ | 112.0 | 106.7 | R |
| 132^bST^ | 1.3 × 10^5^ ±8.2 × 10^3^ | 2.7 × 10^4^ ±9.4 × 10^3^ | 9.7 × 10^3^ ±4.7 × 10^2^ | 20.5 | S | 7.4 | S | 1.3 × 10^5^ ±2.1 × 10^4^ | 1.2 × 10^5^ ±1.7 × 10^4^ | 1.7 × 10^5^ ±9.4 × 10^4^ | 97.4 | 131.6 | R |
| 132^aST^ | 7.7 × 10^4^ ±1.7 × 10^4^ | 2.0 × 10^4^ ±8.2 × 10^3^ | 9.0 × 10^2^ ±8.2 × 10^1^ | 26.1 | S | 1.2 | S | 9.7 × 10^4^ ±1.7 × 10^4^ | 9.0 × 10^4^ ±2.2 × 10^4^ | 9.3 × 10^4^ ±9.4 × 10^3^ | 93.1 | 96.6 | R |
| 133 | 6.7 × 10^4^ ±2.1 × 10^4^ | 1.7 × 10^2^ ±9.4 × 10^1^ | 8.8 × 10^1^ ±9.5 × 10^0^ | 0.3 | S | 0.1 | S | 8.3 × 10^4^ ±1.2 × 10^4^ | 1.6 × 10^5^ ±2.6 × 10^4^ | 1.7 × 10^5^ ±9.4 × 10^4^ | 196.0 | 200.0 | R |
| 133^bST^ | 2.7 × 10^4^ ±1.7 × 10^4^ | 7.5 × 10^1^ ±3.6 × 10^1^ | 8.1 × 10^1^ ±2.5 × 10^1^ | 0.3 | S | 0.3 | S | 9.0 × 10^4^ ±2.2 × 10^4^ | 1.1 × 10^5^ ±1.4 × 10^4^ | 1.3 × 10^5^ ±4.7 × 10^4^ | 122.2 | 148.1 | R |
| 133^aST^ | 2.3 × 10^4^ ±1.2 × 10^4^ | 9.0 × 10^4^ ±8.2 × 10^3^ | 9.4 × 10^1^ ±8.0 × 10^0^ | 385.7 | **R** | 0.4 | S | 4.7 × 10^5^ ±1.2 × 10^5^ | 5.6 × 10^5^ ±3.3 × 10^4^ | 8.0 × 10^5^ ±8.2 × 10^4^ | 120.0 | 171.4 | R |
| 134 | 1.4 × 10^5^ ±4.3 × 10^4^ | 7.3 × 10^4^ ±1.7 × 10^4^ | 7.7 × 10^3^ ±1.7 × 10^3^ | 52.4 | **R** | 5.5 | S | 1.3 × 10^5^ ±4.7 × 10^4^ | 2.1 × 10^5^ ±7.1 × 10^4^ | 2.7 × 10^5^ ±4.7 × 10^4^ | 155.0 | 200.0 | R |
| 134^bST^ | 2.0 × 10^5^ ±2.4 × 10^4^ | 2.0 × 10^5^ ±7.5 × 10^4^ | 1.4 × 10^5^ ±3.7 × 10^4^ | 100.0 | **R** | 70.0 | **R** | 1.8 × 10^5^ ±4.1 × 10^4^ | 1.7 × 10^5^ ±4.5 × 10^4^ | 2.0 × 10^5^ ±7.1 × 10^4^ | 92.6 | 111.1 | R |
| 134^aST^ | 9.0 × 10^4^ ±2.2 × 10^4^ | 1.1 × 10^5^ ±9.4 × 10^3^ | 1.3 × 10^5^ ±2.9 × 10^4^ | 125.9 | **R** | 144.4 | **R** | 6.3 × 10^4^ ±2.6 × 10^4^ | 1.4 × 10^5^ ±2.9 × 10^4^ | 1.3 × 10^5^ ±1.7 × 10^4^ | 221.1 | 200.0 | R |
| 135 | 1.4 × 10^5^ ±3.1 × 10^4^ | 1.2 × 10^5^ ±2.6 × 10^4^ | 2.3 × 10^4^ ±4.7 × 10^3^ | 86.0 | **R** | 16.3 | S | 1.3 × 10^5^ ±2.6 × 10^4^ | 1.5 × 10^5^ ±2.4 × 10^4^ | 2.0 × 10^5^ ±8.2 × 10^4^ | 115.8 | 157.9 | R |
| 135^bST^ | 1.3 × 10^5^ ±2.4 × 10^4^ | 1.2 × 10^4^ ±2.1 × 10^3^ | 2.2 × 10^2^ ±6.1 × 10^1^ | 9.5 | S | 0.2 | S | 1.2 × 10^5^ ±4.7 × 10^3^ | 1.2 × 10^5^ ±1.2 × 10^4^ | 1.3 × 10^5^ ±4.7 × 10^4^ | 100.0 | 108.1 | R |
| 135^aST^ | 1.5 × 10^5^ ±4.1 × 10^4^ | 9.0 × 10^3^ ±1.6 × 10^3^ | 8.3 × 10^1^ ±1.6 × 10^1^ | 6.0 | S | 0.1 | S | 1.3 × 10^5^ ±4.7 × 10^4^ | 1.4 × 10^5^ ±4.5 × 10^4^ | 2.5 × 10^5^ ±4.1 × 10^4^ | 102.5 | 185.0 | R |

Definitions of abbreviations: NHS, normal human serum; HIS, heat-inactivated normal human serum; CFU, colony forming units; RP, resistance pattern; S, sensitive; R, resistant; bST, before the test of stability; aST, after the test of stability
